# Themes and ideologies in China’s diplomatic discourse – a corpus-assisted discourse analysis in China’s official speeches

**DOI:** 10.3389/fpsyg.2023.1278240

**Published:** 2023-11-30

**Authors:** Mingze Liu, Jiale Yan, Guangyuan Yao

**Affiliations:** ^1^College of Marxism, Jilin Normal University, Siping, China; ^2^Irvine Valley College, Irvine, CA, United States; ^3^Department of English, Faculty of Arts and Humanities, University of Macau, Macau, Macau SAR, China

**Keywords:** diplomatic discourse, corpus-assisted discourse analysis, China’s official speeches, critical discourse analysis, China

## Abstract

Diplomatic discourse is a formalized form of political communication that significantly influences a country’s international perception. However, there is a research gap in the analysis of China’s diplomatic discourse, particularly in relation to the speeches available on the official Chinese Foreign Ministry website. This study aims to address this gap by conducting a quantitative and qualitative analysis of China’s diplomatic speeches. This study utilizes a quantitative corpus-assisted discourse analysis to explore the prevalent themes in China’s official speeches. Additionally, qualitative discourse analysis is employed to examine the ideologies manifested in specific examples from the official speeches. The research combines a corpus-based approach with critical discourse analysis to investigate language use, discourse practices, and social practices. The analysis of China’s diplomatic discourse reveals several key themes related to President Xi Jinping’s leadership, international relations, and future community and economy. The findings provide valuable insights into China’s diplomatic strategies and its international image, emphasizing its commitment to cooperation, development, and peace. This research contributes to a better understanding of China’s diplomatic discourse and its role in shaping international perceptions of the country. By highlighting the prevalent themes and ideologies in China’s official speeches, the study emphasizes China’s commitment to fostering positive international relations. The findings offer valuable insights into China’s diplomatic strategies and its efforts to shape its international image.

## 1 Introduction

In recent years, there has been a notable increase in scholarly interest regarding China’s official discourse, particularly during the tenure of President Xi Jinping. Researchers have dedicated their efforts to analyzing the evolution of strategic narratives under his leadership, with a specific focus on how these narratives are strategically tailored to appeal to diverse audiences, both within China and on the international stage (Lams, 2018; Yang, 2020; Brown, 2021). Moreover, scholars have explored the intricate interplay between domestic and international contexts, acknowledging the significant impact of global events and dynamics on China’s diplomatic discourse (Edney, 2014; Chang, 2021; Yang and Chen, 2021).

Previous research has primarily focused on analyzing speeches by President Xi, official conference speeches, and news articles from official outlets (Sun, 2017; Lu, 2018; Lutgard, 2018; Shi and Qu, 2019; Li and Zhu, 2020; Wang, 2021; Zhang et al., 2023). However, there is a significant research gap in the analysis of speeches published on the official Chinese Foreign Ministry website, which encompasses not only the speeches delivered by the Chinese leader but also those delivered by key officials. These speeches, published in English, are considered highly authoritative positions of the Chinese state and are carefully crafted to convey the desired messages to the global community. The study of China’s official discourse, particularly the texts available on the Chinese Foreign Ministry website, provides valuable insights into China’s strategic narratives and diplomatic tools employed to achieve foreign policy objectives. They serve as valuable sources for understanding China’s strategic narratives and the diplomatic tools employed to engage with foreign actors and achieve foreign policy objectives. The study aims to fill the gap in existing research by examining speeches available on the official Chinese Foreign Ministry website and gain insights into China’s official positions, priorities, and strategies. This study could provide a deeper understanding of China’s diplomatic efforts and shed light on its engagement with the international community. It can also contribute to the broader field of diplomatic studies, offering valuable perspectives on the role of discourse in shaping international relations and influencing global perceptions. This research aims to fill this gap by investigating the corpus of speeches found on the aforementioned website.

The following questions will be addressed:

1. What themes are represented in China’s speeches available on the official Chinese Foreign Ministry website?
2. What are the linguistic patterns employed to voice the ideologies in these themes?

## 2 Research design

### 2.1 Corpus

The data utilized in this study was exclusively sourced from the official English website of the Ministry of Foreign Affairs of the People’s Republic of China.^1^ A total of 500 items from the website, spanning from October 29, 2014, to July 4, 2023, were downloaded and saved as text files for further analysis. The title list of all the items can be found in the accompanying document provided in the dataset. The corpus employed for this research is specifically identified as the Corpus of Diplomatic Speeches from the Ministry of Foreign Affairs of the People’s Republic of China (CODS).

### 2.2 Theoretical foundations and analytical framework

The corpus-based approach, as defined by Sinclair (1991), involves the purposeful construction of a collection of natural language texts for specific research purposes. By analyzing authentic text, researchers can uncover previously unknown linguistic characteristics, providing a new perspective on language. This approach allows for the objective observation of regularities in language usage, as researchers can use software to process and analyze data, revealing language use patterns, word lists, concordance lines, collocates, and lexical bundles in the corpus. Thematic analysis can be performed by employing a corpus-based method, which has demonstrated its effectiveness as an analytical technique since the early days of content analysis (Osgood, 1959). Furthermore, contemporary studies have made use of network analysis indicators, as suggested by Danowski (1993). Thematic analysis not only uncovers connections among words but also offers valuable insights into the relationships between words and variables/headings.

One of the advantages of the corpus-based approach is that it offers a comprehensive and reliable qualitative analysis. By analyzing search items, lexical bundles, and collocations in the corpus, researchers can gain insights into the linguistic context and patterns of high-frequency words. This approach minimizes researcher bias, as it relies on natural language data and allows for comparatively objective judgment based on a large amount of data (Baker, 2006). Therefore, corpus-based analysis is often combined with discourse analysis to investigate language use in describing social events.

Critical Discourse Analysis (CDA), on the other hand, takes a critical stance toward language use by examining the underlying ideology and values present in various types of discourse. Fairclough (1992) put forward the theory and method of critical discourse analysis. Compared with traditional discourse analysis, the distinctive feature of critical discourse analysis is that it closely links discourse with social power, reveals social problems through discourse analysis and proposes corresponding strategies to improve the problems on the basis of discourse analysis. Critical discourse analysis is closely linked to Systemic Functional Linguistics (Halliday et al., 2014). As Young and Harrison (2004, 4) suggest, the link between Systemic Functional Linguistics (SFL) and Critical Discourse Analysis (CDA) has a strong tradition. On the one hand, Systemic Functional Linguistics (SFL) has been an important theoretical foundation and linguistic tool for critical discourse analysis (Fowler et al., 1979; Fairclough and Wodak, 1997; Martin and Rose, 2007). On the other hand, Systemic Functional Linguistics (SFL) has been continuously developed in the practice of critical discourse analysis, perfecting the path of SFL in critical discourse analysis. Discourse engages in ideological work (Fairclough and Wodak, 1997; Fairclough, 2010), but ideology is often implicitly embedded in discourse through linguistic means, becoming naturalized common sense, and the operation of ideology is thus difficult to detect. One of the tasks of critical discourse analysis is to reveal the implicit ideological meaning of a discourse through specific linguistic analysis. CDA, as presented by Fairclough (1992), employs a three-dimensional framework that analyzes discourse as text, discourse practice, and social practice. This framework emphasizes that text analysis should not be done in isolation, but rather should consider the broader context in which the discourse is produced and consumed.

The “text” dimension of CDA focuses on the analysis of language in the texts themselves. This involves examining the linguistic qualities and structures present in the discourse. The “discourse practice” dimension addresses the production and consumption of diplomatic speeches, analyzing the strategies employed by news media in shaping and disseminating information. This dimension considers the role of power and ideology in the production and dissemination of discourse. Finally, the “social practice” dimension explores the relationship between discourse practices and the broader social context. It examines the social and cultural practices surrounding the events being reported and how they shape the discourse.

In order to conduct a thorough analysis of the discourse, focusing on the underlying attitude and ideology conveyed in the text, this study adopts Martin and White (2005) framework for appraisal analysis. According to their framework, each segment of text, such as a noun phrase, verb phrase, or clause, that implies an instance of appraisal is referred to as an appraisal group. Within each instance of appraisal, there are typically two primary actors involved: the appraiser and the object of appraisal. The appraisal theory outlined by Martin and White recognizes three key aspects of appraisal between these two actors: Attitude: Attitude represents the emotional essence conveyed by the appraiser toward the object. Various emotions or attitudes such as love, anger, fear, jealousy, excitement, hostility, or satisfaction can be expressed within appraisal groups. Graduation: Graduation pertains to the strength or intensity of the emotion and attitude within each appraisal group. Additionally, the choice of words used to express attitude may also imply a certain level of graduation. Engagement: Sentiments can be expressed directly, indirectly, or attributed to another source. Engagement refers to how speakers or writers express their appraisal or engage in the argument.

### 2.3 Methodology and procedures

By combining corpus linguistics and critical discourse analysis (CDA), this study aims to gain a deeper understanding of language patterns within a specific socio-political context. Corpus linguistics provides the study with efficient methods and tools to analyze a large sample of data, which would be impractical to do manually (Biber et al., 1999; Partington, 2004; Baker et al., 2008). The use of computer-assisted tools allows for the identification of distinct language patterns that may not be easily discernible through qualitative analysis of a small text sample (Morley and Bayley, 2009). On the other hand, critical discourse analysis (CDA) contributes to the study by providing the necessary theories and methods to interpret the data. CDA examines discourse within its socio-historical context and analyzes texts at multiple levels, including topics/themes, discourse strategies, and linguistic means and realizations (Morley and Bayley, 2009; Richards and Schmidt, 2010; Mautner, 2016). This approach allows for a comprehensive analysis of the language patterns and their underlying socio-political factors (Baker, 2006).

To analyze the themes, the study employs the text-mining software KH-Coder, which has been demonstrated to be effective in quantitative content analysis (Higuchi, 2016, 2017). This software performs a co-occurrence network analysis, which identifies high-frequency words in the corpus. The analysis generates a network diagram that visually displays words with similar appearance patterns, connected by lines. This helps to identify the prominent language patterns and their relationships within the corpus.

**Figure 1:**
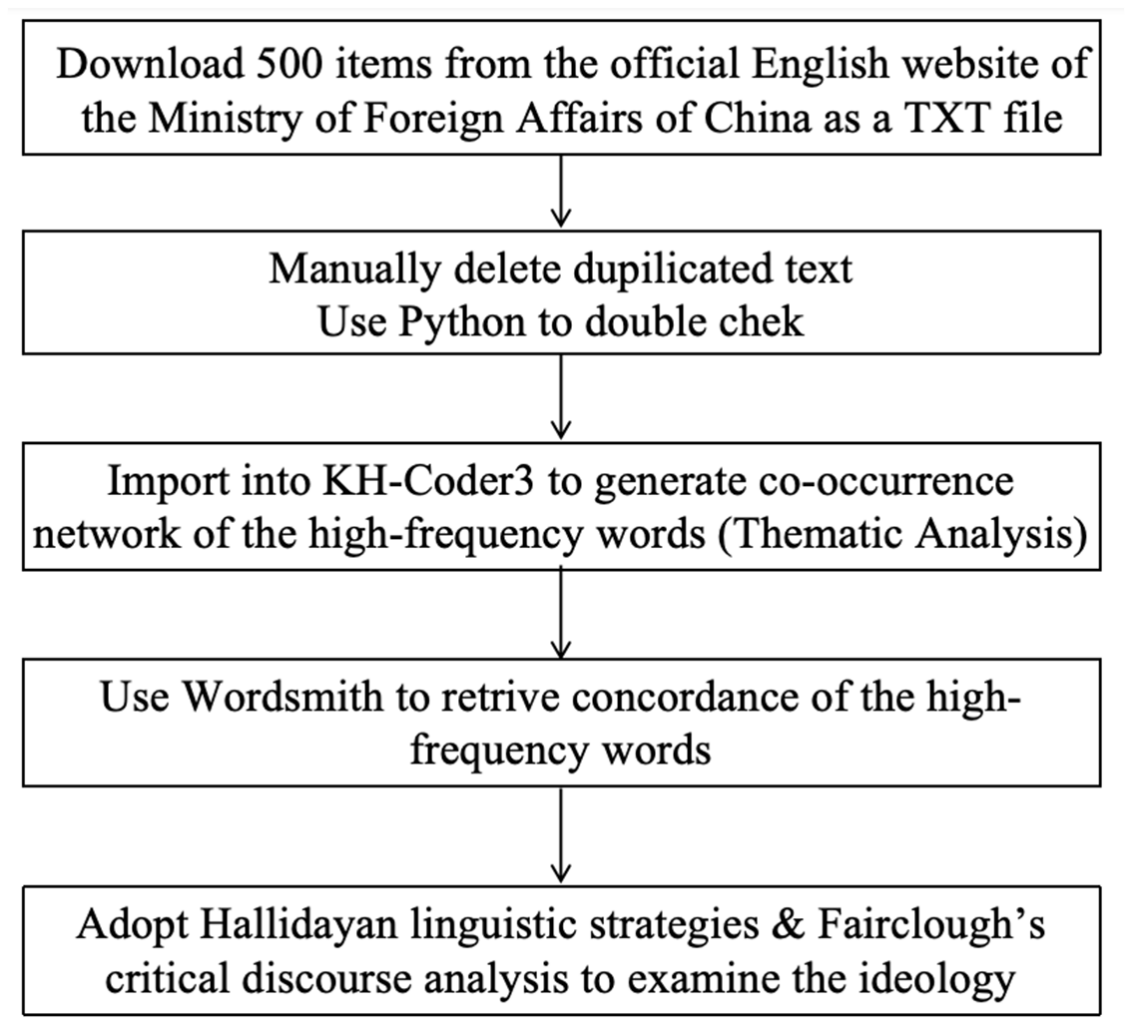
Procedure of the corpus building and analysis.

In addition to KH-Coder, the study also utilizes Wordsmith Tools 7.0, a software that aids in the identification of discursive strategies employed to construct each theme. Through a close analysis of the concordance lines, the study can examine how specific language choices and patterns contribute to the construction of meaning within each theme (Figure 1).

Overall, the combination of corpus linguistics and critical discourse analysis provides a robust framework for analyzing language patterns within a socio-political context. The use of computer-assisted tools enhances the efficiency and accuracy of the analysis, while the theoretical insights from CDA help to interpret and explain the findings. This interdisciplinary approach allows for a comprehensive understanding of the language patterns and their socio-political implications.

## 3 Themes

**Figure 2:**
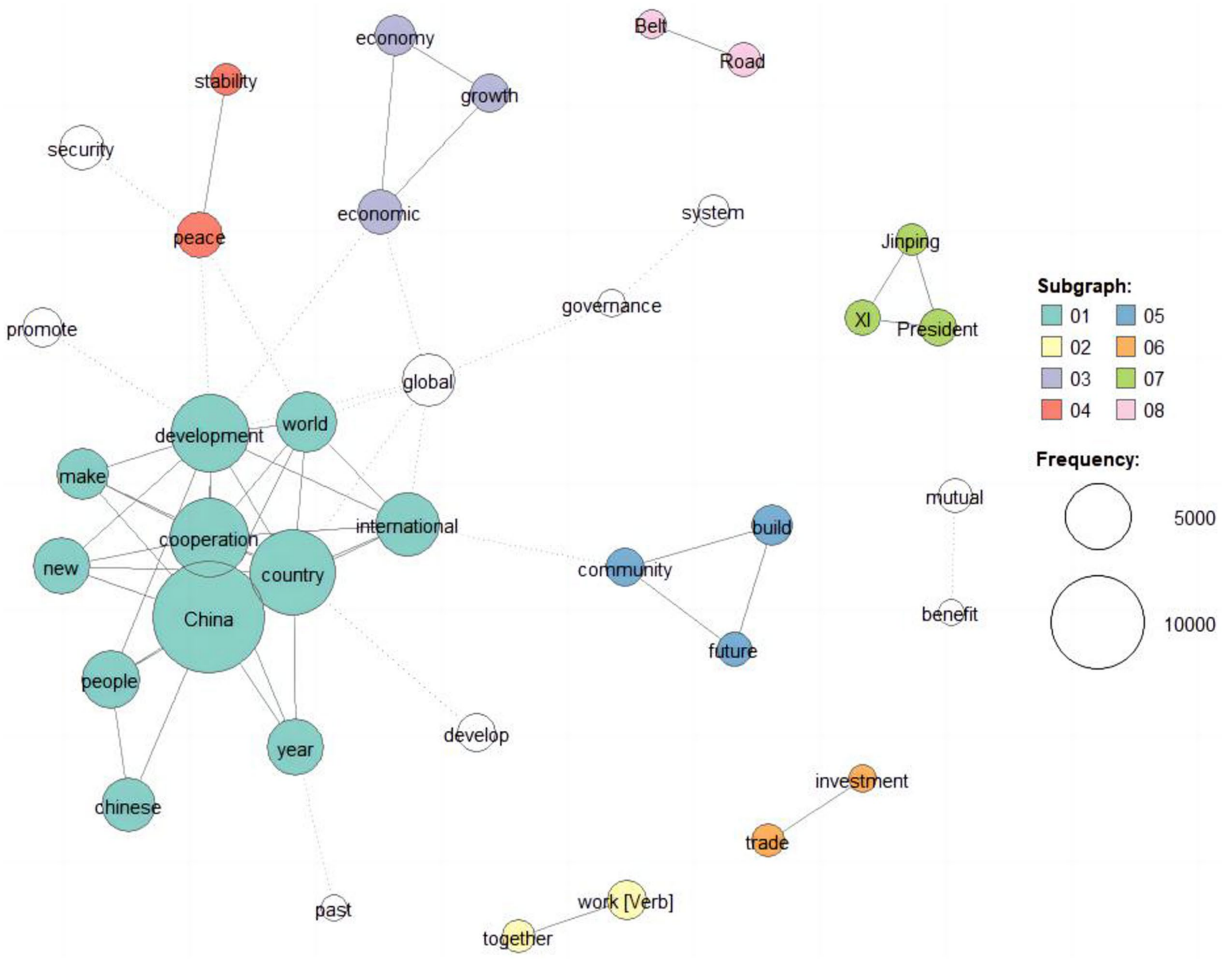
Co-occurrence network of the high-frequency words.

Figure 2 presents an intriguing visualization of the co-occurrence network formed by high-frequency words in the context of CODS. This network analysis reveals that these co-occurring tokens can be categorized into eight distinct clusters, each representing a different thematic focus, which could answer RQ1.

Category 1, as depicted in Figure 2, explores China’s active engagement with the international community and its tireless efforts to foster cooperation and promote global development. This category signifies China’s commitment to playing a constructive role on the world stage and highlights its diplomatic endeavors aimed at forging stronger ties with other nations. Moving on, Category 2 places significant emphasis on the collaborative aspect, emphasizing the importance of working together toward common goals. It underscores the notion that collective efforts and partnerships are crucial for addressing global challenges and achieving shared prosperity. Conversely, Category 3 revolves around the promotion and facilitation of economic growth. The words clustered in this category likely pertain to China’s economic policies, trade relations, and initiatives to enhance economic cooperation, both regionally and globally.

Category 4, as evident from the network, underscores the paramount importance of peace, stability, and security. It suggests that discussions involve themes related to conflict resolution, international security, and the maintenance of peaceful relations among nations. Shifting focus, Category 5 draws attention toward the establishment of a future community. The words clustered in this category may indicate China’s vision of a harmonious and prosperous global community, encompassing diverse nations and cultures, and working toward a shared future. Category 6 delves into the realm of investment and trade, indicating the relevance of economic activities, investment strategies, and trade cooperation within the context under examination. This category likely captures discussions related to China’s role as a major player in the global economy. Category 7, as highlighted by Figure 2, specifically focuses on the role and actions of Chinese President Xi Jinping. This category suggests that President Xi’s leadership and policies are central to the discussions, highlighting his influence and the significance of his decision-making. Lastly, Category 8 delves into the intricacies of the Belt and Road Initiative (BRI). The words clustered in this category likely pertain to China’s ambitious infrastructure and development project, which aims to foster connectivity, trade, and economic cooperation across regions spanning Asia, Europe, Africa, and beyond.

Overall, the co-occurrence network presented in Figure 2 provides valuable insights into the thematic landscape and key areas of focus within CODS. It reveals the multidimensionality of the discussions, ranging from China’s international engagement to economic growth, peace and security, future visions, and the role of President Xi Jinping, as well as the significance of the Belt and Road Initiative.

## 4 Discussion and analysis

The subsequent sections will delve into the seven themes revealed in CODS and conduct an analysis of the language patterns employed, as well as the socio-political factors that underlie them. These themes encompass China’s Engagement with the International Community, Promotion and Facilitation of Economic Growth, Peace, Stability, and Security, Establishment of a Future Community, Investment and Trade, President Xi Jinping, and The Belt and Road Initiative.

### 4.1 China’s engagement with the international community

China has recently become a key provider of development cooperation in the world (Kolmas, 2016; Han, 2017; Chao, 2021). The manifestation of this phenomenon within the discourse can be effectively demonstrated through the explicit acknowledgment of collaborative endeavors involving multiple stakeholders, as well as the comprehensive elucidation of collocations.

#### 4.1.1 Extract 1

This injected strong positive energy of China into the international cooperation on development. It wrote a splendid chapter in the world history of development *(Implement the New Development Agenda and Open Up New Horizons, 2016)*.

Extract 1 exhibits a positive attitude toward China’s contribution to international cooperation on development. The phrase “strong positive energy” implies a favorable evaluation of the impact. The sentence shows a high level of engagement with the topic of international cooperation on development. The discourse contends that China’s contribution as a notable event in the history of development, emphasizing its importance and relevance. In fact, China believes it has the capacity to maintain its position as a prominent force in promoting a diverse global economic structure that benefits both developing and high-income nations in multiple ways (Lin, 2011). This involvement has yielded a multitude of benefits and advancements, fostering collaboration and progress among nations.

#### 4.1.2 Extract 2

We have developed greater synergy between our development strategies and embarked on a path of win-win cooperation with distinctive *features (Two Decades of A Shared Journey toward New Heights in the New Era – Commemorating the 20th Anniversary of The Forum on China-Africa Cooperation, 2020)*.

Extract 2 conveys a positive attitude toward the development strategies and win-win cooperation. The use of phrases like “greater synergy” and “win-win cooperation” implies a favorable evaluation of the outcomes. It demonstrates a high level of engagement with the topic of development strategies and cooperation. It emphasizes the active involvement and effort put into developing the synergy and embarking on the path of win-win cooperation. The sentence generates a sense of optimism and positivity. The mention of “win-win cooperation” indicates mutual benefits and positive outcomes for all parties involved. It conveys the idea of collaboration and shared success. In fact, China’s success can largely be attributed to its effective implementation of “good neighbor diplomacy” strategies, such as the “win-win” approach and policies promoting reform and openness (Womack, 2013).

#### 4.1.3 Extract 3

We need to act with a sense of responsibility and unity, and work together to foster a community of life for man and Nature *(For Man and Nature: Building a Community of Life Together, 2021)*.

#### 4.1.4 Extract 4

China chose not to stand idly by but to work together with other countries to tide over rough times.

Extract 3 conveys a sense of responsibility and urgency. It implies that it is important and necessary for “We” to act and work together. The phrase “with a sense of responsibility and unity” intensifies the evaluation of the desired actions. It emphasizes the need for individuals to take responsibility and unite in their efforts. The sentence evokes a sense of importance and concern. The mention of “fostering a community of life for man and Nature” suggests a caring and nurturing attitude toward the environment and the well-being of both humans and nature. In fact, China’s policies on environmental protection and sustainable development hold significant importance for both China and the global community (Zhang and Wen, 2008). Extract 4 conveys a positive attitude toward China’s proactive approach. The use of phrases like “not to stand idly by” and “work together with other countries” implies a favorable evaluation of the chosen action. The sentence demonstrates a high level of engagement with the topic of cooperation and resilience. It highlights the active involvement of China and its willingness to work alongside other countries. The sentence generates a sense of determination and solidarity. The mention of “tide over rough times” suggests a challenging period or crisis that requires collective action. It conveys a positive emotional impact, emphasizing the resolve to overcome difficulties. In fact, China has established strategic partnerships with nearly a quarter of the countries worldwide (Feng and Huang, 2014).

### 4.2 Promotion and facilitation of economic growth

In the discourse, there is a clear emphasis on the growth of both China’s economy and the global economy. China, as an aspiring economy (Zhao, 2018; Bruton et al., 2021), has been diligently focusing on its economic growth. Also, depending on China’s continued economic growth in the coming decades, China will affect the rest of the world economy (Jin et al., 2016).

#### 4.2.1 Extract 5

It is essential that we keep China’s economic growth on a steady course *(Premier Li Keqiang Meets the Press, 2020)*.

#### 4.2.2 Extract 6

We will do our utmost to keep China’s economic growth stable and make steady progress *(Premier Li Keqiang Meets the Press, 2020)*.

Extract 5 expresses a positive attitude toward the importance of keeping China’s economic growth on a steady course. The word “essential” indicates the speaker’s evaluation that it is crucial for this action to happen. The use of “essential” implies a strong degree of importance or necessity, indicating a high graduation in the evaluation. The speaker positions themselves as being responsible for the action by using the pronoun “we.” This suggests a personal involvement and commitment to the task.

Extract 6 expresses a positive attitude toward the actions of keeping China’s economic growth stable and making steady progress. The phrase “do our utmost” indicates a strong commitment and determination, reflecting a positive evaluation of the speaker’s efforts. The use of “utmost” implies a high degree of effort and commitment, indicating a strong graduation in the evaluation. The speaker positions themselves and others (we) as actively involved in the actions. The phrase “our utmost” shows personal engagement and a sense of responsibility.

Overall, the emphasis on sustaining stable economic growth in China, as highlighted in both Extract 5 and Extract 6, underscores the significance of this objective for the country’s overall development and prosperity. The stability of China’s economic growth is of utmost importance for the country’s overall development and prosperity and also for the whole world (Ding and Tay, 2016). A steady course ensures that the economy remains on track, avoiding sudden fluctuations or downturns that could have adverse effects on various sectors and the livelihoods of the people.

### 4.3 Peace, stability, and security

China collaborates with multiple nations to promote peace and stability in various regions, including Russia, Africa, and others (Moller-Loswick et al., 2015; Adhikari, 2021; Ling, 2021). This commitment is further evident in China’s diplomatic discourse.

#### 4.3.1 Extract 7

At the ninth Beijing Xiangshan Forum held on 22 October 2019, Vice Foreign Minister Le Yucheng pointed out that providing peace, security and a happy life for all the Chinese people, or one fifths of the world’s population, is in itself a major contribution by China to world peace and development *(Le Yucheng: China Is the Safest Country in the World, 2019)*.

Extract 7 expresses a positive attitude toward China’s contribution to world peace and development. It portrays China’s actions as significant and beneficial. The adjective “major” is used to intensify the contribution, indicating the high degree of significance attributed to China’s role in providing peace, security, and a happy life for its people. The statement engages the reader or listener by highlighting the scale of the Chinese population (“one-fifth of the world’s population”), emphasizing the magnitude of the impact and the significance of China’s efforts. The discourse showcases China’s belief that providing peace, security, and a happy life for its people is a significant contribution to world peace and development. It reflects China’s commitment to its citizens’ well-being and its desire to play a constructive role in global affairs. This corresponds to Xi Jinping’s vision of the “Chinese Dream” in relation to Chinese Foreign Policy, wherein the focus has shifted from maintaining a low-key approach to actively displaying and utilizing capabilities while striving for leadership, particularly within the region (Sørensen, 2015).

#### 4.3.2 Extract 8

All in all, China will always be a contributor to world peace, facilitator of global development and proponent of a just and reasonable international order *(Join Hands to Create a Bright Future of Peace and Prosperity, 2016)*.

Extract 8 expresses a positive attitude toward China, portraying it as a consistent contributor to world peace, a facilitator of global development, and a proponent of a just and reasonable international order. It suggests that China plays a significant and positive role in these areas. The adjectives “always” and “just and reasonable” are used to intensify China’s role and the nature of the international order it supports. They convey a strong and unwavering commitment on the part of China. The statement engages the reader or listener by presenting China’s role as an ongoing and continuous contribution to world peace, global development, and a just international order. It implies that China’s actions align with widely shared values and aspirations. It highlights China’s commitment to contributing to world peace, facilitating global development, and advocating for a just and reasonable international order. This statement reflects China seeks to play a more responsible and cooperative role in international affairs (Gill and Huang, 2023).

#### 4.3.3 Extract 9

We will stick to the principle of seeking progress while maintaining stability, ensure the continuity and stability of macro-control policies and adopt pre-adjustment and fine tuning at a proper time within a proper range *(Enhance ASEAN Plus Three cooperation, 2014)*.

Extract 9 expresses a positive attitude toward the principle of seeking progress while maintaining stability. It positions it as a guiding principle and implies that it is valued and considered essential for effective governance. The adjectives “proper” and “fine” are used to qualify the timing and range of the pre-adjustment and fine-tuning of macro-control policies. This suggests a careful and precise approach, indicating an intention to make adjustments within appropriate boundaries. The statement engages the reader or listener by highlighting the commitment to continuity and stability in macro-control policies. It implies a proactive stance toward policy adjustments while ensuring stability and minimizing disruptions (Qin, 2014).

#### 4.3.4 Extract 10

Countries must work together for their national and regional security through dialog and cooperation, and put equal emphasis on development and security, so as to eventually achieve enduring peace and security for all *(China: A Source of Certainty and Stability in a Changing World, 2019)*.

Extract 10 expresses a positive attitude toward collaboration, dialog, cooperation, and the equal emphasis on development and security. It suggests that these actions and approaches are valued and necessary for achieving peace and security. The adjectives “national and regional,” “equal,” and “enduring” are used to intensify the importance and scope of security, development, and peace. They indicate a strong commitment to comprehensive security and long-lasting outcomes. The statement engages the reader or listener by emphasizing the need for countries to work together through dialog and cooperation. It implies that active participation and collaboration are fundamental for achieving the desired peace and security.

Overall, Extract 7 highlights Vice Foreign Minister Le Yucheng’s statement at the Beijing Xiangshan Forum, emphasizing China’s contribution to world peace and development through providing peace, security, and a happy life for its citizens. This reflects China’s belief in the interconnectedness of domestic and global peace and its aspiration to be a positive force in the world (Buzan, 2010). Extract 8 asserts that China will consistently contribute to world peace, facilitate global development, and advocate for a just and reasonable international order. It positions China as a proactive and responsible global actor, committed to maintaining stability, supporting development, and promoting fairness in global governance. Extracts 9 and 10 further emphasize the importance of stability, collaboration, and balancing development and security for achieving enduring peace and security. The discourse portrays China as a committed partner in global affairs, working toward a harmonious and prosperous world (Jisi, 2011).

### 4.4 Establishment of a future community

The People’s Republic of China (PRC) is currently undertaking a global initiative known as the “community of shared future for humankind,” which has the potential to significantly influence the future global order. This concept encompasses multilateral cooperation in various domains, including the economic, political, humanitarian, and security spheres (Ma and Ma, 2018; Semenov and Tsvyk, 2019). This concept is prominently reflected in diplomatic discourse.

#### 4.4.1 Extract 11

China is firmly committed to pursuing the peaceful development path, maintaining the international order with the purposes and principles of the UN Charter at its core, fostering a new type of international relations of win-win cooperation, and building a community of shared future for all mankind *(Jointly Create a Better Future of Peace and Prosperity for Asia Through Dialogue and Consensus, 2016)*.

Extract 11 expresses a positive attitude toward China’s commitment to the mentioned principles and objectives. It positions them as essential and desirable for promoting peace, cooperation, and shared well-being. The adverbs “firmly” and “new” are used to intensify China’s commitment and the nature of the international relations being fostered. They convey a strong and unwavering dedication to these ideals. The statement engages the reader or listener by highlighting China’s active role in pursuing the peaceful development path, maintaining the international order, fostering cooperation, and building a community of shared future. The concept of a community of shared future for all mankind, as advocated by China, emphasizes the importance of global cooperation and mutual benefits for all nations. It promotes the idea that countries should work together to address common challenges and achieve common development (Xiaochun, 2018).

#### 4.4.2 Extract 12

In building a community of shared future for mankind, China encourages all countries to coexist peacefully, engage in sound interaction and seek win-win cooperation *(Work Together to Create a Community of Shared Future for Mankind, 2016)*.

Extract 12 expresses a positive attitude toward the principles of peaceful coexistence, sound interaction, and win-win cooperation. It positions them as desirable and beneficial for fostering a community of shared future for mankind. The adjectives “peacefully,” “sound,” and “win-win” are used to intensify the nature of coexistence, interaction, and cooperation. They suggest a preference for harmonious relationships and mutually beneficial outcomes. The statement engages the reader or listener by emphasizing China’s encouragement for all countries to adopt these principles. It implies an active role in promoting cooperation and a sense of shared responsibility for building a community of shared future.

The discourse constructs China’s image in peaceful coexistence, sound interaction and win-win cooperation. China encourages countries to peacefully coexist, respecting each other’s sovereignty and territorial integrity (Li and Worm, 2011). This principle emphasizes the importance of resolving conflicts through dialog and negotiation rather than resorting to force. Besides, China emphasizes the need for countries to engage in constructive and meaningful interactions (Mazarr et al., 2018). This involves promoting exchanges and cooperation in various fields, such as politics, economy, culture, and science, to foster mutual understanding and trust. Also, China advocates for win-win cooperation, where all parties involved benefit from collaboration (Danzhi, 2019). This principle rejects zero-sum thinking and encourages countries to work together to achieve common development and prosperity.

### 4.5 Investment and trade

China has made substantial investments both in its domestic market and on the international stage. The growth in investments in goods has been remarkable across various countries, particularly in investment-intensive economies like China, since the 1980s (Guo et al., 2021). As a result, China has emerged as a significant source country for outward foreign direct investment flows (Sauvant, 2016).

Since the implementation of its reform and opening-up policies, China has consistently witnessed remarkable growth in its foreign trade (Jin et al., 2016). In recent years, the nation has attained the position of the world’s largest exporter and the second largest importer of goods and commodities. The investment and trade of China have become prominent themes in diplomatic discourse.

#### 4.5.1 Extract 13

China’s door of opening-up will never close. Our policy toward foreign investment will not change. Our protection of legitimate rights and interests of foreign-invested enterprises will not change *(Remarks by H.E. Xi Jinping President of the People’s Republic of China At the Opening Ceremony of the Second World Internet Conference, 2015)*.

Extract 13 expresses a positive attitude toward maintaining an open-door policy and a consistent approach toward foreign investment. It positions these policies as essential and valuable for promoting cooperation and protecting the interests of foreign-invested enterprises. The phrases “never close” and “will not change” intensify the commitment and assurance expressed in the statement. They convey a strong sense of permanence and stability in these policies. The statement engages the reader or listener by emphasizing China’s firm position and assurance regarding the mentioned policies. It implies a sense of reliability in China’s approach to foreign investment (Shan et al., 2012).

#### 4.5.2 Extract 14

President Xi has made clear repeatedly that the door of reform and opening up will not close, but will only open wider and wider. We welcome foreign investment to China. At the same time, we also encourage Chinese companies to explore the global market *(Transcript of Vice Foreign Minister Le Yucheng’s Exclusive Interview with the Financial Times, 2018)*.

Extract 14 expresses a positive attitude toward the door of reform and opening up, foreign investment, and Chinese companies exploring the global market. It positions them as essential and beneficial for promoting economic growth, international cooperation, and global engagement. The phrases “not close,” “open wider and wider,” and “welcome” intensify the commitment, expansion, and openness expressed in the statement. They convey a sense of continuous progress and inclusivity. The statement engages the reader or listener by referencing President Xi’s repeated emphasis and the welcoming attitude toward foreign investment and the encouragement for Chinese companies (Shan et al., 2012). It implies an active role in promoting collaboration, economic development, and global integration.

#### 4.5.3 Extract 15

We have pursued the free trade strategy at a faster pace to build a free trade network with focus on China’s neighbors and covering the whole world *(2015, A Year of Flying Colors for Pursuing Major-Country Diplomacy with Distinctive Chinese Features – Speech by Foreign Minister Wang Yi, 2015)*.

For the trade, Extract 15 expresses a positive attitude toward the pursuit of a free trade strategy and the goal of building a free trade network. It positions them as important and beneficial for promoting economic integration, regional cooperation, and global trade. The mention of pursuing the free trade strategy at a “faster pace” indicates an intensification in China’s efforts. It suggests an increased commitment and urgency in achieving the desired goals. The statement engages the reader or listener by emphasizing China’s active pursuit of the free trade strategy and the goal of building a comprehensive network. It implies a proactive role in promoting economic cooperation and connectivity (Wolff, 2016).

#### 4.5.4 Extract 16

We need to promote global trade and investment to generate growth and build an open world economy *(Remarks by H.E. Xi Jinping President of the People’s Republic of China On the 2016 G20 Summit in China At the Working Lunch of the G20 Summit, 2015)*.

Extract 16 expresses a positive attitude toward the promotion of global trade and investment. It positions them as essential and beneficial for generating economic growth and establishing an open world economy. It suggests that these actions are seen as important drivers of prosperity and global economic integration. The use of the word “need” implies a sense of urgency and importance in promoting global trade and investment. The statement engages the reader or listener by presenting a call to action to promote global trade and investment. It implies an active role for individuals, organizations, or governments in facilitating these processes. It encourages participation and involvement in building a more interconnected and open economy.

### 4.6 President Xi Jinping

Since assuming the position of General Secretary of the Communist Party of China (CPC) in 2012, Xi Jinping has overseen a significant series of political transformations within the country. Under his leadership, the status of Xi Jinping as the Core Leader of the Party has been instrumental in reinforcing the principles of centralized and unified governance within the Central Committee of the CPC (CPCCC). This has led to a notable enhancement of the Party’s leadership structure and its cohesiveness (Guo, 2020). The leadership of President Xi Jinping can be seen in the discourse.

#### 4.6.1 Extract 17

Under the strong leadership of the CPC Central Committee with Comrade Xi Jinping at its core, the “acceleration button” was pressed in China’s modernization drive *(Chinese Modernization: New Opportunities for the World, 2023)*.

Extract 17 expresses a positive attitude toward the leadership of the CPC Central Committee with Comrade Xi Jinping at its core. It positions them as strong and effective in driving China’s modernization efforts. The use of the term “acceleration button” suggests a proactive and decisive approach. The phrase “acceleration button” indicates an intensification or escalation in China’s modernization drive. It implies a deliberate and focused effort to expedite progress. The statement engages the reader or listener by highlighting the central role of the CPC Central Committee, with Comrade Xi Jinping at its core, in pressing the acceleration button. It implies a proactive and hands-on approach to drive China’s modernization.

#### 4.6.2 Extract 18

It was a choice by history and by the people. With the conviction and responsibility of “serving the people selflessly,” President Xi Jinping is steering Chinese modernization forward and leading us in marching on the right path toward a better future *(Chinese Modernization: New Opportunities for the World, 2023)*.

Extract 18 expresses a positive attitude toward President Xi Jinping’s leadership and his role in steering Chinese modernization. It positions him as a dedicated and responsible leader who serves the people selflessly. It suggests that his leadership is seen as crucial for achieving a better future for China. The use of phrases like “choice by history and by the people” and “marching on the right path toward a better future” implies a sense of importance, progress, and positive evaluation of President Xi Jinping’s leadership. The statement engages the reader or listener by emphasizing the significance of President Xi Jinping’s leadership and the sense of responsibility he carries. It suggests that his leadership is actively guiding Chinese modernization and leading the nation toward a better future.

The discourse strategies used in these extracts highlight the central and influential role of President Xi Jinping in driving China’s modernization efforts. The language emphasizes his strong leadership and the collective support of the Communist Party of China (CPC) Central Committee. The use of metaphors and positive language portrays President Xi as a selfless leader dedicated to serving the Chinese people and leading the country on the right path toward a better future. Overall, the discourse aims to reinforce President Xi’s authority and the legitimacy of his leadership in guiding China’s modernization journey (Li, 2016).

### 4.7 The belt and road initiative

The Belt and Road Initiative (BRI) represents China’s most significant international economic endeavor, with the goal of promoting economic development across a vast region. This initiative is designed to reshape China’s external sector and sustain its robust growth. In addition to its focus on infrastructure development, the BRI encompasses a comprehensive range of activities, such as policy dialog, unimpeded trade, financial support, and people-to-people exchange (Huang, 2016; Liu and Dunford, 2016). The term “Belt and Road Initiative” has consistently featured in the discourse.

#### 4.7.1 Extract 19

The Belt and Road initiative is proposed by China, but it is not a “patent” exclusively owned by China. On the contrary, we see it as a symphony and team performance instead of a solo or one-man show. And I am very pleased to see that “One Belt, One Road” has grown to become the shared efforts and aspiration of all populace along its path *(Building the Maritime Silk Road of the 21st Century with Open Mind and Bold Courage, 2015)*.

Extract 19 expresses a positive attitude toward the Belt and Road Initiative and its collective nature. It suggests that the BRI is not exclusively owned by China but is seen as a collaborative effort. The use of terms like “symphony” and “team performance” implies a sense of harmony and collaboration among participating countries. The statement includes graduation markers such as “not exclusively owned” and “shared efforts and aspiration.” It suggests that the perspective presented is a departure from the notion of exclusive ownership and emphasizes the collective nature of the BRI. The statement engages the reader or listener by inviting them to perceive the Belt and Road Initiative as a collaborative endeavor rather than a solo effort. It emphasizes the participation and contributions of multiple countries along the BRI path. It seeks to foster a sense of inclusivity and shared aspirations.

#### 4.7.2 Extract 20

We believe that all partners along the routes of the Belt and Road, whether coastal countries or landlocked ones, could make unique contributions to promoting connectivity and international trade, and as equal contributors to and beneficiaries of this initiative, we should all enjoy the rights to participate in the international maritime cooperation *(Building the Maritime Silk Road of the 21st Century with Open Mind and Bold Courage, 2015)*.

Extract 20 expresses a positive attitude toward the partners along the Belt and Road routes and their potential contributions to connectivity and international trade. It positions them as equal contributors and beneficiaries of the initiative. It also emphasizes the belief in the rights of all partners to participate in international maritime cooperation. The statement includes graduation markers such as “unique contributions,” “equal contributors and beneficiaries,” and “rights to participate.” It suggests that the perspectives presented are seen as important, significant, and deserving of recognition. The statement engages the reader or listener by inviting them to consider the potential of all partners along the Belt and Road routes and their entitlement to participate in international maritime cooperation. It emphasizes the importance of inclusivity and equal opportunities for all partners.

The discourse strategies used in these extracts highlight the collaborative and inclusive nature of the Belt and Road Initiative (BRI). The Belt & Road Initiative represents China’s most significant international economic endeavor, with the goal of promoting economic growth across a vast region that spans sub-regions in Asia, Europe, and Africa (Huang, 2016). The language emphasizes that the initiative is not solely owned by China but is a collective effort involving multiple countries. The use of metaphors, such as “symphony” and “team performance,” portrays the BRI as a collaborative endeavor rather than a one-man show. The discourse also emphasizes the belief that all countries along the BRI routes, regardless of their geographical location, have the potential to contribute to connectivity and international trade. It suggests that all partners should be considered equal contributors and beneficiaries of the initiative, with the right to participate in international maritime cooperation. Overall, the discourse aims to promote a sense of shared ownership and equal participation in the BRI, highlighting its potential benefits for all involved countries.

## 5 Conclusion

Diplomacy is a fundamental aspect of international relations, involving the management of relations between sovereign states (Berridge and Lloyd, 2012). It is through diplomacy that countries engage with one another, negotiate agreements, resolve conflicts, and promote their national interests on the global stage. China, as a major global power, recognizes the importance of diplomacy in shaping its international image and influencing global perceptions. The country’s diplomatic efforts are aimed at projecting a positive image, promoting its interests, and enhancing its standing in the international community. China’s diplomacy is not limited to bilateral relations with other countries, but also extends to multilateral forums such as the United Nations, where it actively participates in global governance and decision-making processes (Li and Hu, 2017).

Diplomatic discourse is a crucial component of China’s diplomatic efforts. It refers to the language and practices employed by the Chinese government to articulate its international strategies and foreign policies (Li and Hu, 2017). This discourse encompasses a wide range of communication channels, including official diplomatic documents, speeches by national leaders, treaties, agreements, communiques, declarations, statements, and press conferences (Afzaal et al., 2019, 2022). Diplomatic discourse is characterized by its formality and authority. It is the most official and authoritative means of international communication, reflecting the government’s diplomatic priorities and objectives (Wu and Cao, 2011). Through diplomatic discourse, China aims to effectively communicate its policies, intentions, and actions to the international community. It seeks to shape international perceptions, garner support, and influence public opinion in its favor (Jin, 2007).

By engaging in diplomatic discourse, China seeks to build alliances, strengthen partnerships, and promote cooperation with other countries. It also aims to address conflicts and disputes through peaceful means, emphasizing the importance of dialog and negotiation (Du and Mei, 2019). Furthermore, diplomatic discourse serves as a platform for China to assert its role as a responsible global actor. It allows the country to showcase its commitment to international norms, principles, and rules, while also advocating for its own interests (Liu and Yang, 2019; Niu and Relly, 2021). Through diplomatic discourse, China seeks to contribute to global governance, promote regional stability, and address global challenges such as climate change, poverty, and terrorism.

Chinese diplomatic discourse plays a crucial role in shaping China’s international image and promoting its interests on the global stage. It serves as a means for China to convey its positions and attitudes on various issues, allowing the country to engage with other nations and influence international perceptions. China has actively utilized its diplomatic discourse to project itself as a peace-loving nation, a victim of foreign aggression, a socialist country, a stronghold of revolution, an anti-hegemonic force, a developing country, a major power, an international collaborator, and an autonomous actor (Wang, 2003). These narratives have been carefully crafted to align with China’s strategic objectives and to garner support from the international community. By enhancing its diplomatic discourse, China can effectively communicate its ideas, policies, and actions to the international community, thereby shaping international perceptions and gaining support for its initiatives.

This study on Chinese diplomatic speeches is in line with previous scholarly works that underscore the significance of enhancing China’s diplomatic capability and refining its discursive framework (Sun, 2020; Sun and Kong, 2020). However, this study uncovers several crucial themes. China’s engagement with the international community is characterized by its active participation in global development cooperation, its efforts to foster cooperation and promote development, and its commitment to maintaining stable economic growth. China’s role as a key provider of development cooperation has been widely recognized, and its injection of positive energy into international cooperation on development has made a significant impact. The collaborative nature of China’s endeavors is emphasized, highlighting the importance of working together to facilitate economic growth and achieve mutual benefits. China’s commitment to peace, stability, and security is evident in its active collaboration with other nations to promote global peace and stability. The concept of a community of shared future for all mankind reflects China’s belief in the importance of global cooperation and mutual benefits. China’s investments and trade initiatives, along with its commitment to an open-door policy, contribute to economic growth and international collaboration. President Xi Jinping’s leadership plays a crucial role in driving China’s modernization efforts and guiding the nation toward a better future. The Belt and Road Initiative, as a collaborative endeavor, aims to promote connectivity, international trade, and cooperation among participating countries. It represents China’s commitment to fostering economic development across a vast region and reshaping its external sector. The initiative encompasses various activities, including policy dialog, unimpeded trade, financial support, and people-to-people exchange.

The analysis of Chinese diplomatic discourse provides valuable insights into China’s diplomatic strategies and its international image. It highlights China’s commitment to cooperation, development, and peace, as well as its efforts to create a better future for all. These findings have implications for China’s diplomatic policies and its interactions with the international community.

Future research could further analyze specific speeches or documents within the corpus to gain a deeper understanding of China’s diplomatic discourse. Comparative studies with other countries’ diplomatic discourses could also be conducted to explore similarities and differences in their approaches and strategies. Additionally, examining the reception and impact of China’s diplomatic discourse on the international stage could provide further insights into its effectiveness and influence.

In conclusion, the analysis of Chinese diplomatic discourse sheds light on China’s engagement with the international community, its commitment to cooperation and development, its emphasis on peace and stability, and its efforts to shape a better future. These findings contribute to our understanding of China’s diplomatic strategies and its role in the global arena.

## Data availability statement

The original contributions presented in the study are included in the article/Supplementary material, further inquiries can be directed to the corresponding author.

## Author contributions

ML: Funding acquisition, Writing – review & editing. JY: Funding acquisition, Writing – review & editing. GY: Writing – original draft.

## Footnotes

^1^ https://www.fmprc.gov.cn/eng/wjdt_665385/zyjh_665391/
