# Supplementary material for: Themes and ideologies in China’s diplomatic discourse – a corpus-assisted discourse analysis in China’s official speeches
Source: Front Psychol. 2023 Nov 30;14:1278240. doi: 10.3389/fpsyg.2023.1278240 (PMC10720904; doi:10.3389/fpsyg.2023.1278240)
Supplement: Supplementary file 1 [file Data_Sheet_1.docx]

Title List of the Data Used

These are the titles of the data we retrieved on the official English website of the Ministry of Foreign Affairs of the People's Republic of China [https://www.fmprc.gov.cn/eng/wjdt_665385/zyjh_665391/].

1. APEC Beijing: China is Ready (Speech by Foreign Minister Wang Yi at the Lanting Forum) (2014-10-29)
2. Let Us Join Hands to Promote Security and Prosperity of Afghanistan and the Region (2014-10-31)
3. Opening Remarks by Foreign Minister Wang Yi At the 26th APEC Ministerial Meeting (2014-11-07)
4. Closing Remarks by H.E. Xi Jinping President of the People's Republic of China At the 22nd APEC Economic Leaders' Meeting At the 22nd APEC Economic Leaders' Meeting (2014-11-11）
5. Remarks by H.E. Li Keqiang Premier of the State Council of the People's Republic of China At the 17th ASEAN-China Summit (2014-11-14)
6. Enhance ASEAN Plus Three cooperation (2014-11-14)
7. Promoting Innovative Development Achieving Interconnected Growth (2014-11-15)
8. Jointly Pursue Dream of Development for China and Australia and Realize Prosperity and Stability in Our Region (2014-11-17)
9. Laying the Foundations of Peace and Stability for An Asian Community of Shared Destiny (2014-11-21)
10. Remarks by Mr. Xu Hong, Director-General of the Department of Treaty and Law of the Ministry of Foreign Affairs, on the Position Paper of the Government of the People's Republic of China on the Matter of Jurisdiction in the South China Sea Arbitration Initiated by the Republic of the Philippines (2014-12-07)
11. Remarks by Assistant Foreign Minister Zhang Kunsheng at the ARF Seminar on Sea Lines of Communications Security (2014-12-08)
12. Toast by Foreign Minister Wang Yi at New Year Reception for 2015 (2014-12-11)
13. Chinese President Xi Jinping's 2015 New Year Message (2015-01-04)
14. Jointly Write a New Chapter in the Partnership of Comprehensive Cooperation Between China and Latin America and the Caribbean (2015-01-08)
15. Impromptu Remarks by Foreign Minister Wang Yi at the Welcoming Event of the First Ministerial Meeting of the China-CELAC Forum (2015-01-09)
16. Records of Foreign Minister Wang Yi's Interview with CCTV Africa (2015-01-11)
17. Keynote Speech by Foreign Minister Wang Yi At the First Ministerial Meeting of the China-CELAC Forum (2015-01-13)
18. Premier Li Keqiang Holds Dialogue with Representatives of International Business Council of World Economic Forum (2015-01-22)
19. Uphold Peace and Stability, Advance Structural Reform and Generate New Momentum for Development (2015-01-23)
20. Foster a Vision of Common, Comprehensive, Cooperative And Sustainable Security to Build a Better World Of Enduring Peace and Common Development (2015-02-07)
21. Building An East Asia Partnership of Win-Win Cooperation (2015-02-07)
22. Building the Maritime Silk Road of the 21st Century with Open Mind and Bold Courage (2015-02-12)
23. Remarks by Foreign Minister Wang Yi at the Inauguration of The Year of China-Pakistan Friendly Exchanges (2015-02-13)
24. Carry Forward the Purposes and Principles of the UN Charter And Build a New Type of International Relations Featuring Win-win Cooperation (2015-02-24)
25. Foreign Minister Wang Yi Meets the Press (2015-03-08)
26. Premier Li Keqiang Meets the Press (2015-03-16)
27. Toward a New Type of International Relations of Win-Win Cooperation (2015-03-25)
28. Full text of Chinese President's speech at Boao Forum for Asia (2015-03-28)
29. Speech by Foreign Minister Wang Yi at the Session "ASEAN Community: A Major Milestone for Asian Integration" Of the Boao Forum for Asia Annual Conference 2015 (2015-03-28)
30. Jointly Build the 21st Century Maritime Silk Road By Deepening Mutual Trust and Enhancing Connectivity (2015-03-29)
31. Remarks by Vice Minister of Foreign Affairs Li Baodong At the Joint Launch Event for New Chinese Website of The United Nations (2015-04-10)
32. Carry Forward the Bandung Spirit and Advance the International Rule of Law in Today's World (2015-04-13)
33. Strengthen Asia-Africa Solidarity and Cooperation To Uphold World Peace and Justice (2015-04-14)
34. Uphold the Authority of the UN Charter and Promote Win-Win Cooperation (2015-04-16)
35. Transcript of Premier Li Keqiang's Interview with Financial Times Editor Lionel Barber (2015-04-16)
36. Building a China-Pakistan Community of Shared Destiny to Pursue Closer Win-Win Cooperation (2015-04-21)
37. Carry Forward the Bandung Spirit for Win-win Cooperation (2015-04-22)
38. Promote Industrial Cooperation for Common Development (2015-04-28)
39. China's Statement by H.E. Mr. Fu Cong, Ambassador for Disarmament, on Nuclear Disarmament in NPT RevCon (2015-05-04)
40. Jointly Safeguard the Victory of World War Two and Create a Bright Future of Win-Win Cooperation (2015-05-05)
41. EU and China strategic partners for long run (2015-05-07)
42. Promoting China-Latin America Common Development by Upgrading China-Brazil Cooperation (2015-05-21)
43. Remarks by Chinese State Councilor Wang Yong at The Opening Ceremony of ARF Disaster Relief Exercise 2015 (2015-05-26)
44. Toward Win-win Cooperation Through Amity, Sincerity, Mutual Benefit and Inclusiveness (2015-06-12)
45. Director-General Zhang Jun Publishes Article on Collaboration on Production Capacity on China Daily (2015-06-19)
46. China-US Cooperation Benefits the Two Countries and the World (2015-06-23)
47. For Closer Bonds Between Chinese and American People (2015-06-23)
48. Why is it important for China and the United States to engage in Dialogue? (2015-06-23)
49. Li Yuanchao Addresses the Opening Ceremony of the Fourth World Peace Forum (2015-06-27)
50. China's Role in the Global and Regional Order: Participant, Facilitator and Contributor (2015-06-27)
51. China-EU Summit joint statement (2015-06-29)
52. China and Europe: Working Together for New Progress In China-EU Relations (2015-06-29)
53. Li Keqiang Delivers Speech at OECD, Stressing the Focus on Development for Common Prosperity and Promotion of Mutual Benefits and Multi-win Results Through International Cooperation in Production Capacity (2015-07-02)
54. Statement by H.E. Li Baodong Vice Foreign Minister of China At the Vilnius Sherpa Meeting of the Nuclear Security Summit 2016 (2015-07-02)
55. Keep Development in Focus and Create Prosperity for All (2015-07-03)
56. Building Partnership Together Toward a Bright Future (2015-07-09)
57. Strengthening Shanghai Cooperation Organization Through Unity, Mutual Support and Joint Response to Challenges (2015-07-10)
58. Remarks by Vice Foreign Minister Li Baodong At the International Seminar on The 70th Anniversary of the United Nations (2015-07-20)
59. Carry Forward the Tazara Spirit In a Joint Effort to Build a Community of Shared Destiny (2015-07-23)
60. Video Message by Foreign Minister Wang Yi At the Launch Ceremony of The Report on China's Implementation of The Millennium Development Goals (2015-07-24)
61. Address by Vice Foreign Minister Li Baodong At the Launch Ceremony of the Report on China's Implementation Of the Millennium Development Goals (2000-2015) (2015-07-24)
62. Speech at the Reception Commemorating The 70th Anniversary of the Victory of The Chinese People's War of Resistance Against Japanese Aggression And the World Anti-Fascist War (2015-09-03)
63. For China-US Friendly Cooperation, For Global Peace and Development (2015-09-16)
64. Review the Spirit of September 19 Joint Statement and Uphold Peace and Stability of the Korean Peninsula and Northeast Asia (2015-09-19)
65. Yang Jiechi: Jointly Write a New Chapter of China-US Friendship and Cooperation (2015-09-21)
66. Xi Jinping Gives Interview to "The Wall Street Journal", Emphasizing Building New Model of Major-Country Relationship Between China and US and Enhancing Peace, Stability and Development in Asia-Pacific Region and World (2015-09-22)
67. Address by H.E. Xi Jinping President of the People's Republic of China At the China-US Governors' Forum (2015-09-23)
68. Speech by H.E. Xi Jinping President of the People's Republic of China At the Welcoming Dinner Hosted by Local Governments And Friendly Organizations in the United States (2015-09-23)
69. Join Hands to Strengthen Cooperation for the Realization of Common Development (2015-09-25)
70. Remarks by H.E. Xi Jinping President of the People's Republic of China At Arrival Ceremony On the South Lawn of the White House (2015-09-26)
71. Towards Win-win Partnership for Sustainable Development (2015-09-27)
72. Promoting Women's All-round Development and Building a Better World for All Remarks by H.E. Xi Jinping President of the People's Republic of ChinaAt the Global Leaders' Meeting on Gender Equality And Women's Empowerment (2015-09-28)
73. Remarks at the Ceremony of Presenting Zun of Peace from China to the United Nations (2015-09-28)
74. China is Here for Peace (2015-09-28)
75. Working Together to Forge a New Partnership of Win-win Cooperation and Create a Community of Shared Future for Mankind (2015-09-29)
76. Remarks by H.E. Li Keqiang Premier of the State Council At Reception Marking the 66th Anniversary of The Founding of the People's Republic of China (2015-09-30)
77. To Build a New Middle East of Peace, Tolerance and Self-advancement (2015-10-01)
78. Video Message by Foreign Minister Wang Yi At the Opening Ceremony of the Third Arctic Circle Assembly (2015-10-17)
79. Keynote Speech by Vice Foreign Minister Zhang Ming at the China Country Session of the Third Arctic Circle Assembly (2015-10-17)
80. Uphold Win-Win Cooperation and Promote Peace and Stability in the Asia-Pacific (2015-10-17)
81. Build Asia-Pacific Consensus, Implement Cooperation Agenda and Develop Regional Economy (2015-10-19)
82. Congratulatory Message by President Xi Jinping to the Opening Ceremony of the 9th UNESCO Youth Forum (2015-10-26)
83. For a New Chapter of the China-ROK Friendship (2015-10-31)
84. Keynote Speech by Premier Li Keqiang at the Welcoming Luncheon Hosted by the Business Community of the Republic of Korea (2015-11-02)
85. Keep China-Japan-ROK Cooperation in the Right Lane (2015-11-02)
86. Remarks by H.E. Li Keqiang Premier of the State Council of the People's Republic of China At the Closing Ceremony of the China-France Economy and Climate Summit (2015-11-03)
87. Address by H.E. Li Yuanchao Vice President of the People's Republic of China At the Reception of the 60th Anniversary Of China-Afghanistan Diplomatic Relations (2015-11-04)
88. Promote China-Afghanistan Cooperation and Bring New Life to the Silk Road (2015-11-05)
89. Join Hands to Deliver a Bright Future for China-Vietnam Relations (2015-11-05)
90. To Renew the Friendship Across High Mountains and Contribute Asian Wisdom to World Peace and Development (2015-11-06)
91. Work Together to Write a New Chapter of China-Vietnam Friendship (2015-11-06)
92. Build on Past Achievements to Create a Bright Future For China-Singapore Relations (2015-11-07)
93. Forging a Strong Partnership to Enhance Prosperity of Asia (2015-11-07)
94. Innovative Growth That Benefits All (2015-11-16)
95. Expand Opportunities and Meet Challenges Together (2015-11-16)
96. Asia-Pacific Free Trade Area: The Road to Better Economic Integration in the Region (2015-11-17)
97. Taking forward the APEC Agenda (2015-11-17)
98. Remarks by H.E. Xi Jinping President of the People's Republic of China On the 2016 G20 Summit in China At the Working Lunch of the G20 Summit (2015-11-17)
99. China, G20 and Global Governance (2015-11-18)
100. The Leading Role of the Asia-Pacific In Meeting Global Economic Challenges (2015-11-18)
101. Deepening Partnership for a Prosperous Asia-Pacific (2015-11-19)
102. Remarks by H.E. Li Keqiang Premier of the State Council of the People's Republic of China At the 18th China-ASEAN Summit (2015-11-22)
103. Remarks by H.E. Li Keqiang Premier of the State Council of the People's Republic of China At the 10th East Asia Summit (2015-11-23)
104. Remarks by H.E. Li Keqiang Premier of the State Council of the People's Republic of China At the Fourth Summit of China and Central and Eastern European Countries (2015-11-25)
105. Work Together to Open Up New Prospects for Win-Win Cooperation (2015-11-25)
106. Build on Past Achievements and Open up the Future of All-round Development of China-Africa Friendship and Cooperation (2015-11-26)
107. Work Together to Build a Win-Win, Equitable and Balanced Governance Mechanism on Climate Change (2015-11-30)
108. Speech by Vice Foreign Minister Li Baodong at The Opening Ceremony of The Third ASEAN Regional Forum Workshop on Space Security (2015-11-30)
109. Let the Flower of China-Zimbabwe Friendship Bloom with New Splendor (2015-11-30)
110. Remarks by Executive Vice Minister Zhang Yesui At the Opening Ceremony of the First BRICS Media Summit (2015-12-01)
111. Working Together to Write a New Chapter In China-Africa Cooperation (2015-12-04)
112. Open a New Era of China-Africa Win-Win Cooperation and Common Development (2015-12-04)
113. A Rainbow of Friendship and Cooperation with Greater Splendor (2015-12-05)
114. Solidarity for Security and Cooperation for Development (2015-12-09)
115. 2015: A Year of Flying Colors for Pursuing Major-Country Diplomacy with Distinctive Chinese Features (2015-12-12)
116. Remarks by H.E. Li Keqiang Premier of the State Council of the People's Republic of China At the Extended Session of the 14th Meeting of The Council of Heads of Government of Member States of The Shanghai Cooperation Organization (2015-12-15)
117. Remarks by H.E. Xi Jinping President of the People's Republic of China At the Opening Ceremony of the Second World Internet Conference (2015-12-16)
118. Remarks at the ARF Seminar on Maritime Risks Management and Cooperation (2015-12-18)
119. Give Peace A Chance and People Tranquility (2015-12-19)
120. Chinese President Xi Jinping's 2016 New Year Message (2015-12-31)
121. Remarks at Foreign Ministry' 2016 New Year Reception (2016-01-06)
122. Strengthen Partnership for a Better Future (2016-01-15)
123. Address by President Xi Jinping of China At the Opening Ceremony of The Asian Infrastructure Investment Bank (2016-01-16)
124. Foreign Minister Wang Yi Gives Interview to Xinhua News Agency on "Implementation Day" of Joint Comprehensive Plan of Action on Iranian Nuclear Issue (2016-01-17)
125. Full text of Chinese President's signed article on Saudi newspaper (2016-01-18)
126. Full text of Chinese President's signed article on Egyptian newspaper (2016-01-20)
127. Full text of Chinese president's signed article on Iranian newspaper (2016-01-21)
128. Implement the New Development Agenda and Open Up New Horizons (2016-01-21)
129. Provide New Driving Forces for Innovative Development of the World Economy (2016-01-22)
130. China-Latin America Overall Cooperation Sets Sail (2016-01-29)
131. A Changing China and Its Diplomacy (2016-02-26)
132. An Open China:From Ningxia to the World (2016-03-02)
133. Address by H.E. Li Keqiang Premier of the State Council of the People's Republic of China At the First Lancang-Mekong Cooperation Leaders' Meeting (2016-03-23)
134. A New Vision for A Dynamic Asia Through Joint Efforts (2016-03-24)
135. China Remains Committed to Peaceful Settlement of Disputes in the South China Sea through Negotiations and Consultations (2016-03-25)
136. Remarks by Foreign Minister Wang Yi at the G20 Session of The Boao Forum for Asia Annual Conference 2016 (2016-03-25)
137. Deepening Regional Cooperation for a Community of Shared Future (2016-03-25)
138. Full text of Chinese President Xi Jinping's article on Czech newspaper (2016-03-26)
139. Strengthen Global Nuclear Security Architecture and Promote Global Nuclear Security Governance (2016-04-02)
140. China's Development and China-Europe Cooperation: Sources of Positive Energy for the World (2016-04-14)
141. Deepen Mutual Trust and Coordination and Work Together for Regional Security and Development (2016-04-28)
142. Jointly Create a Better Future of Peace and Prosperity for Asia Through Dialogue and Consensus (2016-04-28)
143. Toast by Assistant Foreign Minister Qian Hongshan at the Reception Celebrating the 20th Anniversary of ASEM (2016-05-10)
144. Remarks by Assistant Foreign Minister Qian Hongshan at The Opening Ceremony of the ASEM Media Dialogue on Connectivity (2016-05-10)
145. Briefing by XU Hong, Director-General of the Department of Treaty and Law on the South China Sea Arbitration Initiated by the Philippines (2016-05-12)
146. Speech by H.E. Mr. Liu Zhenmin Vice Minister of Foreign Affairs of China At the Photo Exhibition of The China-AALCO Exchange and Research Program On International Law (2016-05-18)
147. Remarks by Assistant Foreign Minister Qian Hongshan At the Belt and Road Side Event Of the 72nd Session of the United Nations Economic and Social Commission for Asia and the Pacific (2016-05-19)
148. Transcript of Foreign Minister Wang Yi's Interview With Belahodood of Al Jazeera (2016-05-19)
149. Remarks by Assistant Foreign Minister Qian Hongshan At the General Debate of the 72nd Session of the United Nations Economic and Social Commission for Asia and the Pacific (2016-05-19)
150. Remarks by Vice President Li Yuanchao at the Reception In Honor of President Pranab Mukherjee (2016-05-26)
151. Remarks by Foreign Minister Wang Yi At the Media Briefing on the G20 Hangzhou Summit (2016-05-26)
152. An Open China: Guangxi Embracing the World (2016-05-27)
153. Work Together to Create a Community of Shared Future for Mankind (2016-05-31)
154. The Minutes of the Meeting Between Vice Foreign Minister Liu Zhenmin and US Media Delegation on the South China Sea Issue (2016-06-03)
155. Remarks by State Councilor Yang Jiechi At the Closing Session and Joint Press Availability of the Eighth Round of China-US Strategic and Economic Dialogue (2016-06-07)
156. Remarks by Vice Premier Wang Yang At the Closing Session and Press Conference of The Eighth Round of the China-US Strategic and Economic Dialogue (2016-06-07)
157. Making Unremitting Efforts for a New Model of Major-Country Relationship Between China and the United States (2016-06-07)
158. Statement by Vice Foreign Minister Li Baodong Head of the Chinese Delegation at The Ministerial Meeting of the Twentieth Anniversary of the Comprehensive Nuclear-Test-Ban Treaty (2016-06-13)
159. Li Keqiang's speech at Summer Davos opening ceremony (2016-06-29)
160. Build a New Type of International Relations Featuring Win-Win Cooperation (2016-07-01)
161. Speech by Dai Bingguo at China-US Dialogue on South China Sea Between Chinese and US Think Tanks (2016-07-05)
162. Actively Practice the Asian Security Concept and Jointly Create a New Future of Asia-Pacific Security (2016-07-12)
163. Remarks by Chinese Foreign Minister Wang Yi on the Award of the So-called Arbitral Tribunal in the South China Sea Arbitration (2016-07-12)
164. Vice Foreign Minister Liu Zhenmin at the Press Conference on the White Paper Titled China Adheres to the Position of Settling Through Negotiation the Relevant Disputes Between China and the Philippines in the South China Sea (2016-07-13)
165. Yang Jiechi Gives Interview to State Media on the So-called Award by the Arbitral Tribunal for the South China Sea Arbitration (2016-07-15)
166. Join Hands to Create a Bright Future of Peace and Prosperity (2016-07-16)
167. Statement by Vice Foreign Minister Li Baodong At the General Debate of the Ministerial Meeting of High-Level Political Forum on Sustainable Development (2016-07-21)
168. Statement by Vice Foreign Minister Li Baodong At Voluntary National Review of the Implementation of 2030 Agenda for Sustainable Development (2016-07-21)
169. Work Report by Foreign Minister Wang Yi At the Plenary Session of the Coordinators' Meeting On the Implementation of the Follow-up Actions of The Johannesburg Summit of The Forum on China-Africa Cooperation (2016-07-29)
170. President Xi Jinping's Congratulatory Message and State Councilor Yang Jiechi's Keynote Speech to The Opening Ceremony of the Coordinators' Meeting on the Implementation of The Follow-up Actions of the Johannesburg Summit of The Forum on China-Africa Cooperation (2016-07-29)
171. Keynote Speech by H.E. Xi Jinping, President of the People's Republic of China, at the Opening Ceremony of the B20 Summit (2016-09-03)
172. Yang Jiechi Gives Interview on G20 Hangzhou Summit (2016-09-07)
173. Deepen Production Capacity Cooperation, Achieve Common Development (2016-09-12)
174. Work Together for a World of Peace, Stability and Sustainable Development (2016-09-22)
175. Realize Win-Win Cooperation in Asia By Promoting Exchange and Mutual Learning among Civilizations (2016-10-10)
176. Building a Stronger ACD for an Asian Community of Shared Future (2016-10-11)
177. Work together to improve regional security architecture and address common challenges (2016-10-12)
178. Statement by Ambassador WANG Qun, Director-General of the Arms Control Department of the Ministry of Foreign Affairs of China, at the General Debate of the First Committee of the 71st Session of the UNGA (2016-10-13)
179. Speech by H.E. State Councilor Yang Jiechi At the Reception for the 45th Anniversary of the Restoration of The Lawful Seat of the People's Republic of China in the United Nations (2016-10-26)
180. The Asia-Pacific Economic Integration Forebodes a Bright Future (2016-11-16)
181. Work Together to Embrace the Bright Future of China-US Business Cooperation (2016-11-23)
182. Speech by Foreign Minister Wang Yi at the Opening of the Symposium On International Developments and China's Diplomacy in 2016 (2016-12-03)
183. China is Committed to a Human Rights Development Path With Chinese Characteristics (2016-12-10)
184. Forging Sound Relations through the Principle of Amity, Sincerity, Mutual Benefit and Inclusiveness, Add New Chapters in Neighborhood Diplomacy (2017-01-10)
185. Jointly Shoulder Responsibility of Our Times, Promote Global Growth (2017-01-17)
186. Remarks by H.E. Yang Jiechi State Councilor of the People's Republic of China At the Opening Ceremony of 2017 First BRICS Sherpa Meeting (2017-02-23)
187. Transcript of Premier Li Keqiang's Meeting with the Press at the Fifth Session of the 12th National People's Congress (2017-03-16)
188. Work Together to Build Partnerships and Pursue Peace and Development (2017-03-20)
189. Steadily Promote Cooperation among South China Sea Coastal States (2017-03-25)
190. Deepen Regional Cooperation in Asia With Renewed Confidence (2017-03-26)
191. Director-General Zhang Jun Published Article Entitled "China's Economic Diplomacy Entered the New Era" (2017-04-19)
192. Stay Committed to the Goal of Denuclearization Uphold Peace and Stability on the Peninsula (2017-04-29)
193. Statement by H.E. Mr. Fu Cong,Head of Chinese Delegation,Ambassador for Disarmament Affairs of the People's Republic of China at the General Debate in the First Session of the Preparatory Committee for the 2020 NPT Review Conference (2017-05-02)
194. Full text of President Xi's speech at opening of Belt and Road forum (2017-05-15)
195. Work Together to Fight Poverty and Achieve Common Prosperity (2017-06-21)
196. Working Together to Address Global Security Challenges and Build a Community of Shared Future for Mankind (2017-06-24)
197. Speech by Executive Vice Foreign Minister Zhang Yesui At the Luncheon of the Sixth World Peace Forum (2017-06-26)
198. Transcript of Premier Li Keqiang's Dialogue With WEF Chief and International Business Leaders At the Annual Meeting of the New Champions 2017 (2017-06-28)
199. Keynote Speech at the Opening Ceremony of 15th East Asia Forum (2017-06-30)
200. Study and Implement General Secretary Xi Jinping'sThought on Diplomacy in a Deep-going Way andKeep Writing New Chapters of Major-Country Diplomacy with Distinctive Chinese Features (2017-07-17)
201. Forge Ahead under the Guidance of General Secretary Xi Jinping's Thought on Diplomacy (2017-09-01)
202. Toward Peace and Development for All (2017-09-21)
203. Fulfilling a Serious Commitment to a Future of Peace (2017-09-21)
204. Press release Meeting of BRICS Ministersof Foreign Affairs/International Relations (2017-09-26)
205. Speech by H.E. Li Keqiang Premier of the State Council of the People's Republic of China At the 20th ASEAN Plus China, Japan and ROK Summit (2017-11-14)
206. Speech by H.E. Li Keqiang Premier of the State Council of the People's Republic of China At the 20th China-ASEAN Summit (2017-11-14)
207. Speech by H.E. Li Keqiang Premier of the State Council of the People's Republic of China At the 12th East Asia Summit (2017-11-15)
208. Speech by H.E. Li Keqiang Premier of the State Council of The People's Republic of China At the Sixth Summit of Heads of Government of China and Central and Eastern European Countries (2017-11-28)
209. Address by H.E. Li Keqiang Premier of the State Council of the People's Republic of China at the Seventh China-CEEC Business Forum (2017-11-28)
210. Writing a New Chapter of International Human Rights Exchanges and Cooperation (2017-12-10)
211. Speech by Foreign Minister Wang Yi at the Opening of Symposium on International Developments and China's Diplomacy in 2017 (2017-12-10)
212. Advance the Global Human Rights Cause and Build a Community with a Shared Future for Mankind (2017-12-11)
213. Join Hands Across the Ocean in a New Era (2018-01-25)
214. Pursue High-quality Development,Work Together for Global Economic Prosperity and Stability (2018-01-25)
215. Director-General of the Department of InternationalEconomic Affairs of Foreign Ministy Elaborated onChina's Contributions to World Economy (2018-01-25)
216. Remarks by Foreign Minister Wang Yi at the Opening Ceremony of China-CELAC Economic and Trade Cooperation Forum and China-LAC Business Council Annual Meeting 2018 (2018-01-27)
217. Remarks by H.E. Wang Yi Minister of Foreign Affairs of the People's Republic of China At the 2018 New Year Reception (2018-01-30)
218. Foreign Minister Wang Yi Meets the Press (2018-03-09)
219. Premier Li Keqiang Meets the Press (2018-03-21)
220. Vice Foreign Minister Zheng Zeguang Addresses the 19th Annual Session of the China Development Forum (2018-03-26)
221. Remarks by H.E. Wang Yi State Councilor and Minister of Foreign Affairs of The People's Republic of China at the GMS Business Summit (2018-03-30)
222. Working Together to Write a New Chapter of Sub-regional Cooperation (2018-03-31)
223. Keynote Speech by H.E. Li Keqiang Premier of the State Council of the People's Republic of China At the China-Indonesia Business Summit (2018-05-08)
224. Keynote Address by H.E. Li Keqiang Premier of the State Council of the People's Republic of China At ASEAN Secretariat (2018-05-08)
225. Address by Assistant Foreign Minister Chen Xiaodong at The Opening Ceremony of the Seventh China-Africa Think Tank Forum (2018-07-04)
226. Working Together to Build a World of Lasting Peace and Universal Security and a Community with a Shared Future for Mankind (2018-07-14)
227. A Joint Response to the Big Change of the World and A Joint Effort to Build a Community with a Shared Future (2018-07-14)
228. Full text of Chinese President Xi's signed article on UAE media (2018-07-18)
229. Full text of Chinese President Xi's signed article on Senegalese media (2018-07-20)
230. Address by H.E. Li Keqiang Premier of the State Council of the People's Republic of China At the Opening Ceremony of The Annual Meeting of the New Champions 2018 (2018-09-20)
231. Transcript of Premier Li Keqiang's Dialogue with WEF Chief and International Business Leaders At the Annual Meeting of the New Champions 2018 (2018-09-21)
232. Commemorate Nelson Mandela and Join Hands for Lasting Peace (2018-09-25)
233. Transcript of Vice Foreign Minister Le Yucheng's Exclusive Interview with the Financial Times (2018-09-26)
234. Opening a New Chapter of UN Peacekeeping to Honor Our Shared Commitments to Peace (2018-09-26)
235. Working Together to Fulfill Our Noble Responsibility for Common Peace and Security (2018-09-27)
236. Seize the Historic Opportunity for Peace on the Korean Peninsula (2018-09-28)
237. Multilateralism, Shared Peace and Development (2018-09-29)
238. Promote and Protect Human Rights in the Process of Development (2018-09-30)
239. An opportune time for closer Asia-Europe cooperation: new challenges and opportunities (2018-10-17)
240. Remarks by H.E. Yang Jiechi at the Foreign Policy Session of The 2018 Annual Meeting of the Valdai Discussion Club (2018-10-18)
241. Speech by H.E. Wang Yi State Councilor and Minister of Foreign AffairsAt the Opening of Symposium on The International Situation and China's Foreign Relations in 2018 (2018-12-11)
242. Remarks by Executive Vice Foreign Minister Le YuchengAt the Welcoming Dinner ofThe First Meeting of the Advisory Council ofThe Belt and Road Forum for International Cooperation (BRF) (2018-12-21)
243. Remarks by H.E. Wang Yi State Councilor and Minister of Foreign Affairs At the MFA 2019 New Year Reception (2019-01-31)
244. Remarks by Mr. Fu Cong,Director General of Department of Arms Control at the Dialogue between the P5 Delegations and Academia (2019-02-02)
245. Keynote Speech by Assistant Foreign Minister Chen Xiaodong at the Dialogue on the Implementation of China-Africa Peace and Security Initiative (2019-02-07)
246. Meeting People's Aspiration for a Better Life Through Continued Progress on Human Rights in China (2019-03-15)
247. Yang Jiechi on the Belt and Road Initiative and Preparations for the Second Belt and Road Forum for International Cooperation (2019-03-30)
248. Yang Jiechi on the Belt and Road Initiative and Preparations for the Second Belt and Road Forum for International Cooperation (2019-03-30)
249. Toast by H.E. Xi Jinping President of the People's Republic of China At the Welcoming Banquet of The Second Belt and Road Forum for International Cooperation (2019-04-26)
250. Working Together to Deliver a Brighter Future For Belt and Road Cooperation (2019-04-26)
251. Remarks by H.E. Xi Jinping President of the People's Republic of China At the Press Conference of The Second Belt and Road Forum for International Cooperation (2019-04-27)
252. Promoting High-Quality Development of Belt and Road Cooperation (2019-04-27)
253. Working Together for a Green and Better Future for All (2019-04-28)
254. Deepening Exchanges and Mutual Learning Among Civilizations For an Asian Community with a Shared Future (2019-05-15)
255. Remarks by Assistant Foreign Minister Zhang Jun at the 75th Session of the United Nations Economic and Social Commission for Asia and the Pacific (2019-05-27)
256. Advancing Global Sustainable Development Through High-quality Belt and Road Cooperation (2019-05-27)
257. Written Interview by H.E. Xi Jinping President of the People's Republic of China With Mainstream Russian Media Organizations (2019-06-05)
258. Assistant Foreign Minister Zhang Jun Publishes a Signed Article on Jointly Strengthening Global Strategic Stability between China and Russia (2019-06-12)
259. Working Together for a Brighter Future of China-Tajikistan Friendship (2019-06-12)
260. Staying Focused and Taking Solid Actions For a Brighter Future of the Shanghai Cooperation Organization (2019-06-14)
261. Working Together for New Progress of Security and Development in Asia (2019-06-15)
262. Remarks by H.E. Yang Jiechi Member of the Political Bureau of the CPC Central Committee and Director of the Office of the Central Commission for Foreign Affairs At the Group Meeting with the Heads of African Delegations Attending the Coordinators' Meeting on the Implementation of The Follow-up Actions of the FOCAC Beijing Summit (2019-06-24)
263. Keynote Speech by H.E. Wang Yi State Councilor and Foreign Minister of The People's Republic of China At the Opening Ceremony of the Coordinators' Meeting on The Implementation of the Follow-up Actions of The Beijing Summit of the Forum on China-Africa Cooperation (2019-06-25)
264. Working Together to Build a High-Quality World Economy (2019-06-28)
265. Address by H.E. Li Keqiang Premier of the State Council of The People's Republic of China At the Opening Ceremony of The Annual Meeting of the New Champions 2019 (2019-07-04)
266. China's Foreign Policy in a Fast Changing World:Mission and Responsibility (2019-07-08)
267. Upholding Peace and Cooperation and Building a Community with a Shared Future for Mankind (2019-07-09)
268. Promoting Asian Cooperation for a Shared Bright Future (2019-08-07)
269. General Statement by H.E. Ambassador Li Song at the Fifth Conference of State Parties to the Arms Trade Treaty (2019-08-28)
270. Premier Li Keqiang's Written Interview With Russian News Agency TASS (2019-09-15)
271. China in the New Era: Innovative Hunan Embracing the World (2019-09-19)
272. A Joint Response to Climate Change A Better Environment for Our Planet (2019-09-24)
273. Toward a Better Future Through Development Cooperation (2019-09-25)
274. Vision and Conviction Will Take China-US Relations Forward (2019-09-25)
275. Statement by H.E. Mr. Fu Cong Head of the Chinese Delegation and Director-General of the Department of Arms Control of MFA At the 2019 Conference on Facilitating the Entry into Force of the Comprehensive Nuclear-Test-Ban Treaty (2019-09-26)
276. China Today: A Proud Member of the Global Community (2019-09-28)
277. Speech by Xi Jinping At the Reception in Celebration of the 70th Anniversary of The Founding of the People's Republic of China (2019-09-30)
278. Remarks by H.E. Li Keqiang Premier of the State Council of the People's Republic of China At the Closing Ceremony of The International Horticultural Exhibition 2019 Beijing China (2019-10-10)
279. Statement by Ambassador FU Cong, Director-General of the Department of Arms Control of the Ministry of Foreign Affairs of China, at the General Debate of the First Committee of the 74thSession of the UNGA (2019-10-12)
280. Maintaining Global Strategic Stability, Reducing Risks of Nuclear Conflicts (2019-10-16)
281. Remarks by Director General FU Cong at the Opening Session of the Third Training Course for the 1540 Points of Contact in the Asia-Pacific Region (2019-10-21)
282. China: A Source of Certainty and Stability in a Changing World (2019-10-22)
283. Le Yucheng: The World Needs a New Vision of Security (2019-10-22)
284. Le Yucheng: Why This Path Has Worked for China (2019-10-22)
285. Le Yucheng: China Cannot Develop by Simply Taking Advantage of Others (2019-10-22)
286. Le Yucheng: China Will Continue to Resolutely Safeguard Its National Security and Core Interests (2019-10-22)
287. Le Yucheng: China Is the Safest Country in the World (2019-10-22)
288. Working Together to Meet Our Shared Responsibility And Build a Community with a Shared Future for Mankind (2019-11-01)
289. Speech by H.E. Li Keqiang Premier of the State Council of The People's Republic of China At the 18th Meeting of the Council of Heads of Government of Member States of the Shanghai Cooperation Organization (2019-11-03)
290. Speech by H.E. Li Keqiang Premier of the State Council of The People's Republic of China At the 22nd China-ASEAN Summit (2019-11-04)
291. Speech by H.E. Li KeqiangPremier of the State Council ofThe People's Republic of ChinaAt the 14th East Asia Summit (2019-11-05)
292. Speech by H.E. Li KeqiangPremier of the State Council ofThe People's Republic of ChinaAt the 22nd ASEAN Plus China, Japan and ROK Summit (2019-11-05)
293. Openness and Cooperation for a Shared Future (2019-11-05)
294. Remarks by H.E. Mr. Fu Cong, Director-General of the Department of Arms Control of the Ministry of Foreign Affairs of China on "The Future of Arms Control and Non-Proliferation Regime" at the 2019 Moscow Non-Proliferation Conference (2019-11-08)
295. Remarks by H.E. Mr. Fu Cong Director-General of the Department of Arms Control of the Ministry of Foreign Affairs of China on "JCPOA: Possible Steps to Prevent Further Escalation and Sustain the Deal" at the 2019 Moscow Nonproliferation Conference (2019-11-10)
296. Remarks by H. E. Wang Qishan Vice President of the People's Republic of China At the Second Paris Peace Forum (2019-11-13)
297. Together for a New Chapter in BRICS Cooperation (2019-11-15)
298. Beautiful Qinghai -- From the Origin of the Three Rivers to the World (2019-11-27)
299. Braving Waves and Sailing Forward with Resolve (2019-12-13)
300. Enhancing Mutual Trust and Cooperation To Embrace an Even Better Future of China-EU Relations (2019-12-17)
301. Adhering to Multilateralism and Jointly Creating a Better Future (2019-12-17)
302. Building on 70 Years of Achievements and Pursuing Progress in the New Era (2019-12-24)
303. Remarks by H.E. Li Keqiang Premier of the State Council of the People's Republic of China At the Eighth Trilateral Summit Meeting Among The People's Republic of China, the Republic of Korea and Japan (2019-12-25)
304. Written Interview with Al-Ahram by State Councilor and Foreign Minister Wang Yi (2020-01-08)
305. Full text of Xi's signed article on Myanmese newspapers (2020-01-16)
306. Remarks by H.E. Xi Jinping President of the People's Republic of China At Launch Ceremony of Celebrations for 70th Anniversary of China-Myanmar Diplomatic Relations & China-Myanmar Year of Culture and Tourism (2020-01-18)
307. Remarks by H.E. Wang Yi State Councilor and Minister of Foreign Affairs of The People's Republic of China At the MFA 2020 New Year Reception (2020-01-20)
308. Transcript of State Councilor and Foreign Minister Wang Yi's Exclusive Interview with Reuters (2020-02-15)
309. Bringing the East and West Together In Shared Commitment to Multilateralism (2020-02-15)
310. Working Together to Defeat the COVID-19 Outbreak (2020-03-26)
311. President Xi Jinping Attends Extraordinary G20 Leaders' Summit and Delivers Important Remarks (2020-03-27)
312. Deepening BRICS Cooperation to Combat COVID-19 (2020-04-28)
313. Carrying Forward the Shanghai Spirit To Safeguard the Multilateral System (2020-05-13)
314. Message of Congratulations from H.E. Wang Yi State Councilor and Minister of Foreign Affairs of The People's Republic of China to the Reopening of the Embassy of the People's Republic of China in the Republic of Kiribati (2020-05-15)
315. Fighting COVID-19 Through Solidarity and Cooperation Building a Global Community of Health for All (2020-05-18)
316. Premier Li Keqiang Meets the Press: Full Transcript of Questions and Answers (2020-05-28)
317. Defeating COVID-19 with Solidarity and Cooperation (2020-06-17)
318. Adhering to the Path of Solidarity, Cooperation and Win-win Results (2020-06-19)
319. Remarks by State Councilor and Foreign Minister Wang Yi At the Video Conference of RIC Foreign Ministers (2020-06-23)
320. The Trend Toward China-US Cooperation is Unstoppable (2020-07-08)
321. Stay on the Right Track and Keep Pace with the Times to Ensure the Right Direction for China-US Relations (2020-07-09)
322. Study and Implement Xi Jinping Thought on Diplomacy Conscientiously and Break New Ground in Major-Country Diplomacy with Chinese Characteristics (2020-07-20)
323. Remarks by H.E. Xi Jinping President of the People's Republic of China At the Opening Ceremony of the Fifth Annual Meeting of The Asian Infrastructure Investment Bank (2020-07-28)
324. Interview on Current China-US Relations Given by State Councilor and Foreign Minister Wang Yi to Xinhua News Agency (2020-08-06)
325. Respect History, Look to the Future andFirmly Safeguard and Stabilize China-US Relations (2020-08-07)
326. Reviving the Cold War is Anachronistic (2020-08-12)
327. Speech by H.E. Li Keqiang Premier of the State Council ofThe People's Republic of China At the Third Lancang-Mekong Cooperation Leaders' Meeting (2020-08-24)
328. Upholding the Trend of Peace and Development of Our World with Unity, Cooperation, Openness and Inclusiveness (2020-08-31)
329. Upholding Multilateralism and Moving in the Right Direction of Human Progress (2020-09-02)
330. Jointly Safeguarding Peace, Stability and Development in the South China Sea with Dialogue, Consultation and Win-Win Cooperation (2020-09-02)
331. Strengthening Cooperation to Weather the Trying Times (2020-09-04)
332. Upholding Multilateralism, Fairness and Justice and Promoting Mutually Beneficial Cooperation (2020-09-08)
333. Remarks by H.E. Wang Yi State Councilor and Minister of Foreign Affairs of The People's Republic of China At the Video Opening Ceremony of Intra-Afghan Negotiations (2020-09-13)
334. Remarks by Vice Foreign Minister Ma Zhaoxu at Vision China Program (2020-09-16)
335. Interview Given by State Councilor and Foreign Minister Wang Yi to Xinhua News Agency (2020-09-17)
336. Message of Congratulations from H. E. Wang Yi State Councilor and Minister of Foreign Affairs of the People's Republic of China for the Opening of the Embassy of the People's Republic of China in Solomon Islands (2020-09-21)
337. Remarks by H.E. Xi Jinping President of the People's Republic of China At the High-level Meeting to Commemorate the 75th Anniversary of the United Nations (2020-09-21)
338. Statement by H.E. Xi Jinping President of the People's Republic of China At the General Debate of the 75th Session of The United Nations General Assembly (2020-09-22)
339. Deepening CICA Cooperation and Upholding Multilateralism (2020-09-25)
340. Improving the Global Governance System Through Reform and Joining Hands to Build a Community with a Shared Future for Humankind (2020-09-25)
341. Working Together on Poverty Eradication,Promoting New Progress of South-South Cooperation (2020-09-26)
342. The Only Viable Choice for the International Community:Strengthening Solidarity and Cooperation, Rejecting Confrontation and Division (2020-09-28)
343. Statement by H.E. Xi Jinping President of the People's Republic of China At the High-level Meeting on the Twenty-fifth Anniversary of The Fourth World Conference on Women (2020-10-01)
344. Global Vision and Firm Commitment as a Major Country (2020-10-02)
345. Two Decades of A Shared Journey toward New Heights in the New Era (2020-10-16)
346. Keynote Speech by H.E. Xi Jinping President of the People's Republic of China At the Opening Ceremony of The Third China International Import Expo (2020-11-04)
347. Upholding Multilateralism to Tackle Global Challenges (2020-11-07)
348. Carrying Forward the Shanghai Spirit and Deepening Solidarity and Collaboration for a Stronger Community with a Shared Future (2020-11-10)
349. Build on Twenty Years of Proud Achievements and Open Up a New Chapter in China-Africa Relations (2020-11-12)
350. Standing Together to Fight COVID-19,Promote Recovery and Safeguard Peace (2020-11-12)
351. Statement by Director-General FU Cong at the EU Non-proliferation and Disarmament Conference (2020-11-13)
352. Fighting COVID-19 in Solidarity and Advancing BRICS Cooperation Through Concerted Efforts (2020-11-17)
353. Fostering a New Development Paradigm and Pursuing Mutual Benefit and Win-win Cooperation (2020-11-19)
354. Written Interview with Asharq Al-Awsat by State Councilor and Foreign Minister Wang Yi (2020-11-19)
355. Strengthening Solidarity and Cooperation to Build a Better Future Together (2020-11-20)
356. Working Together for an Asia-Pacific Community with A Shared Future (2020-11-20)
357. Together, Let Us Fight COVID-19 and Create a Better Future (2020-11-21)
358. Remarks by President Xi Jinping at Leaders' Side Event on Safeguarding Planet of G20 Riyadh Summit (2020-11-22)
359. Remarks by H.E. Xi Jinping President of the People's Republic of China At the Opening Ceremony of the 17th China-ASEAN Expo and China-ASEAN Business and Investment Summit (2020-11-27)
360. Speech by H.E. Li Keqiang Premier of the State Council of The People's Republic of China At the 19th Meeting of the Council of Heads of Government of Member States of the Shanghai Cooperation Organization (2020-12-01)
361. Reinforce Global Cooperation with Renewed Solidarity to Defeat COVID-19 (2020-12-04)
362. Building Consensus for Solidarity and Cooperation and Working Together for a Peaceful and Prosperous Africa (2020-12-05)
363. Keynote Address by H.E. Deng Li Assistant Minister of Foreign Affairs of China At the Opening Ceremony of the 14th Senior Officials Meeting of The Forum on China-Africa Cooperation (2020-12-10)
364. Serving the Country and Contributing to the World: China's Diplomacy in a Time of Unprecedented Global Changes and a Once-in-a-Century Pandemic (2020-12-11)
365. Building on Past Achievements and Launching a New Journey for Global Climate Actions (2020-12-13)
366. Reorient and Steer Clear of DisruptionsFor a Smooth Sailing of China-U.S. Relations (2020-12-19)
367. Non-Traditional Security Threats and International Counter-Terrorism Cooperation (2020-12-22)
368. Remarks by State Councilor and Foreign Minister Wang YiAt a Joint Press Availability with Tanzanian Foreign MinisterPalamagamba Kabudi (2021-01-08)
369. Rise to the Challenges, Serve the Nation and Embark on a New Journey for Major-Country Diplomacy with Chinese Characteristics (2021-01-16)
370. Let the Torch of Multilateralism Light up Humanity's Way Forward (2021-01-25)
371. Anything is Possible When China and the United States Choose to Cooperate (2021-01-28)
372. Dialogue with National Committee on U.S.-China Relations (2021-02-02)
373. Remarks by H.E. Luo Zhaohui Vice Minister of Foreign Affairs At the Online Seminar on Ecological Environment Protection of Trans-Himalaya Forum for International Cooperation (2021-02-03)
374. Lunar New Year Message from H.E. Wang Yi State Councilor and Minister of Foreign Affairs of The People's Republic of China (2021-02-07)
375. Remarks by State Councilor and Foreign Minister Wang Yi At the Ninth Ministerial Conference of The Heart of Asia-Istanbul Process (2021-03-30)
376. A Hubei Reborn for New Glories; A China Embracing Openness and Prosperity (2021-04-12)
377. Transcript of Vice Minister Le Yucheng's Exclusive Interview with the Associated Press of the United States (2021-04-18)
378. Pulling Together Through Adversity and Toward a Shared Future for All (2021-04-20)
379. Working in the Same Direction to Build Peace Together (2021-04-20)
380. For Man and Nature: Building a Community of Life Together (2021-04-22)
381. Focusing on Cooperation and Managing Differences:Bringing China-U.S. Relations Back to the Track of Sound and Steady Development (2021-04-24)
382. Remarks by State Councilor and Foreign Minister Wang Yi at the United Nations Security Council High-level Meeting on the Theme "Maintenance of International Peace and Security: Upholding Multilateralism and the United Nations-centered International System" (2021-05-08)
383. End the Fighting and Violence Immediately and Uphold Equity and Justice (2021-05-17)
384. Practice True Multilateralism and Galvanize Strong Support For Africa's Development (2021-05-20)
385. Working Together to Build a Global Community of Health for All (2021-05-21)
386. Staying Open and Inclusive and Upholding Multilateralism:Toward a Community with a Shared Future for Mankind (2021-05-26)
387. Forge ahead Together to Deliver a Brighter Future for China and Africa (2021-05-27)
388. An Impressive Scorecard (2021-06-14)
389. Let Us Strengthen Confidence and Solidarity and Jointly Build a Closer Partnership for Belt and Road Cooperation (2021-06-24)
390. A New Journey Ahead after Fifty Extraordinary Years (2021-06-25)
391. Promote Solidarity and Cooperation In Pursuit of Multilateralism (2021-06-29)
392. Uphold World Peace and Promote Human Progress (2021-07-03)
393. Remarks by H.E. Wang Yi State Councilor and Foreign Minister of the People's Republic of China At the Palestine-Israel Peace Symposium (2021-07-16)
394. Build on Past Achievements and Scale New Heights (2021-07-28)
395. Strengthening Vaccine Cooperation to Build a Great Wall Against COVID-19 (2021-08-06)
396. Remarks by H.E. Xi JinpingPresident of the People's Republic of ChinaAt the Global Trade in Services Summit ofThe 2021 China International Fair for Trade in Services (2021-09-03)
397. Bolstering Confidence and Jointly Overcoming Difficulties To Build a Better World (2021-09-22)
398. Eliminating Racism and Building a World of Equality for All (2021-09-23)
399. Building Consensus and Synergy For a Bright Future of Global Development (2021-09-26)
400. Harnessing Strengths of CICA Cooperation and Building an Asian Community with a Shared Future (2021-10-12)
401. Working Together to Build a Community of All Life on Earth (2021-10-12)
402. Working Together in Support of a Bright Future for Afghanistan (2021-10-13)
403. Staying Connected with the World and Abreast with the Times And Making Big Strides on the Path of Sustainable Development (2021-10-14)
404. Speech by H.E. Xi Jinping President of the People's Republic of China at the Conference Marking the 50th Anniversary of the Restoration of the Lawful Seat of the People's Republic of China in the United Nations (2021-10-25)
405. Acting in Solidarity for a Shared Future (2021-10-30)
406. Let the Breeze of Openness Bring Warmth to the World (2021-11-04)
407. Enhancing Solidarity and Cooperation to Build Together A Maritime Community with a Shared Future (2021-11-09)
408. Pursuing Sustainable Development in a Concerted Effort To Build an Asia-Pacific Community with a Shared Future (2021-11-11)
409. Opening a New Chapter Together For Asia-Pacific Economic Cooperation (2021-11-12)
410. China-U.S. Presidential Meeting:Setting Direction and Providing Impetus for Bilateral Relations (2021-11-16)
411. Uphold the Tradition of Always Standing Together And Jointly Build a China-Africa Community With a Shared Future in the New Era (2021-11-29)
412. Seizing Opportunities to Maintain and Advance China-U.S. Economic and Trade Cooperation and Sub-national Exchange (2021-11-30)
413. Keynote Speech by Vice Foreign Minister Xie Feng  At the Opening Ceremony of  2021 Understanding China Conference (Guangzhou) (2021-12-02)
414. On Democracy, the People Know the Best (2021-12-02)
415. Report by State Councilor and Foreign Minister Wang Yi At the Eighth Ministerial Conference of the Forum on China-Africa Cooperation (2021-12-03)
416. Speech by Vice Minister Ma Zhaoxu at the Workshop on GDI Cooperation towards Accelerated Implementation of the 2030 Agenda (2021-12-09)
417. Remarks by State Councilor Wang Yi ​At the Meeting of the Advisory Council of The Belt and Road Forum for International Cooperation 2021 (2021-12-18)
418. China's Diplomacy in 2021: Embracing a Global Vision and Serving the Nation and its People (2021-12-20)
419. Adapt to Changes in International Counterterrorism Landscape and Join Hands to Write a New Chapter for Counterterrorism Cooperation (2021-12-23)
420. State Councilor and Foreign Minister Wang Yi Gives Interview to Xinhua News Agency and China Media Group on International Situation and China’s Diplomacy in 2021 (2021-12-30)
421. 2022 New Year address by President Xi Jinping (2021-12-31)
422. Striding Forward Holding High the Banner of Building a Community with a Shared Future for Mankind (2022-01-01)
423. Upholding China-Philippines Friendship,Jointly Creating a Brighter Future (2022-01-17)
424. Forge Ahead with Confidence and Fortitude to Jointly Create a Better Post-COVID World (2022-01-17)
425. ​Lunar New Year Message from H.E. Wang Yi  State Councilor and Minister of Foreign Affairs of  The People’s Republic of China (2022-01-29)
426. Toast by H.E. Xi Jinping President of the People’s Republic of China At the Welcoming Banquet of The Olympic Winter Games Beijing 2022 (2022-02-05)
427. Drawing Wisdom from History to Light up the Road Ahead (2022-02-28)
428. Upholding Equity and Justice to Promote Sound Development of the Global Human Rights Cause (2022-02-28)
429. Premier Li Keqiang Meets the Press: Full Transcript of Questions and Answers (2022-03-11)
430. ​Cherish Peace, Work Together in Unity, and Pursue a Win-Win Future for the Asia-Pacific (2022-03-19)
431. Remarks by H.E. Wang Yi at the Opening Ceremony of The Session of the Council of Foreign Ministers of the Organization of Islamic Cooperation (2022-03-22)
432. Rising to Challenges and Building a Bright Future Through Cooperation (2022-04-21)
433. Acting on the Global Security Initiative to Safeguard World Peace and Tranquility (2022-04-24)
434. Acting on the Global Security Initiative To Safeguard World Peace and Tranquility (2022-05-06)
435. Address by State Councilor and Foreign Minister Wang Yi At the High-Level Virtual Meeting of the Group of Friends of The Global Development Initiative (2022-05-09)
436. Working Collectively and Pursuing Coordinated Development To Promote Global Desertification Governance (2022-05-10)
437. Studying and Implementing Xi Jinping Thought on Diplomacy in a Deep-going Way and Opening up New Horizons in China's External Work (2022-05-16)
438. Address by H.E. Xi Jinping President of the People’s Republic of China At the Conference of the 70th Anniversary of CCPIT and Global Trade and Investment Promotion Summit (2022-05-18)
439. Jointly Building an Asia-Pacific Community with a Shared Future (2022-05-23)
440. ​Keep Abreast of the Trend of the Times to Shape a Bright Future (2022-06-22)
441. Fostering High-quality Partnership and Embarking on a New Journey of BRICS Cooperation (2022-06-23)
442. Forging High-quality Partnership For a New Era of Global Development (2022-06-24)
443. Working as Cooperation Partners for True Multilateralism (2022-07-10)
444. Upholding Peace, Development, Independence and Inclusiveness And Renewing the Firm Commitment to Open Regionalism (2022-07-12)
445. Carrying Forward the DOC Spirit and Forging Regional Consensus To Jointly Build a Sea of Peace, Friendship and Cooperation (2022-07-25)
446. Upholding the Treaty on the Non-Proliferation of Nuclear Weapons for World Peace and Development (2022-08-03)
447. Vice Foreign Minister Ma Zhaoxu on Pelosi’s Visit to Taiwan (2022-08-09)
448. China and Africa: Strengthening Friendship, Solidarity and Cooperation for a New Era of Common Development (2022-08-19)
449. Implement UNCLOS in Full and in Good Faith and Actively Contribute to Global Maritime Governance (2022-09-02)
450. Late-night Phone Call, Quick Action, and Deep Brotherhood (2022-09-08)
451. Build on the Past to Make Greater Strides in China-Kazakhstan Relations (2022-09-13)
452. Working Together for a Brighter Future of China-Uzbekistan Relations (2022-09-13)
453. Ride on the Trend of the Times and Enhance Solidarity and Cooperation to Embrace a Better Future (2022-09-16)
454. A Trip that Pointed the Way Forward in Turbulent Times and Reinvigorated the Ancient Silk Road (2022-09-17)
455. Jointly Advancing the Global Development Initiative and Writing a New Chapter for Common Development (2022-09-21)
456. The Right Way for China and the United States to Get Along in the New Era (2022-09-23)
457. Making Every Effort for Peace and Development and Shouldering the Responsibility for Solidarity and Progress (2022-09-25)
458. Stay True to the Original Aspiration and Carry Forward the Friendship for a Shared Future (2022-09-24)
459. Meeting Challenges with Stronger Solidarity and Coordination (2022-10-13)
460. Full text of the report to the 20th National Congress of the Communist Party of China (2022-10-25)
461. Speech by H.E. Li Keqiang Premier of the State Council of the People's Republic of China At the 21st Meeting of the Council of Heads of Government of Member States of the Shanghai Cooperation Organization (2022-11-02)
462. Coordinating Security and Development to Advance Ocean Governance (2022-11-03)
463. Working Together for a Bright Future of Openness and Prosperity (2022-11-04)
464. Protecting Wetlands for Our Future and Scaling up Wetlands Action Across the World (2022-11-05)
465. For a Stronger China-Cambodia Friendship and A Closer East Asian Cooperation (2022-11-07)
466. Speech by H.E. Li Keqiang Premier of the State Council of the People’s Republic of China At the 25th China-ASEAN Summit (2022-11-12)
467. Speech by H.E. Li Keqiang Premier of the State Council of the People’s Republic of China At the 25th ASEAN Plus China, Japan, ROK Summit (2022-11-13)
468. Speech by H.E. Li Keqiang Premier of the State Council of the People’s Republic of China At the 17th East Asia Summit (2022-11-14)
469. Working Together to Meet the Challenges of Our Times and Build a Better Future (2022-11-15)
470. Staying Committed to and Jointly Promoting Development to Bring Asia-Pacific Cooperation to New Heights (2022-11-17)
471. Shouldering Responsibility and Working Together in Solidarity To Build an Asia-Pacific Community with a Shared Future (2022-11-18)
472. Full text of Xi Jinping's speech at memorial meeting for Comrade Jiang Zemin (2022-12-06)
473. Building on Past Achievements and Jointly Creating a BrighterFuture of China-GCC Relations (2022-12-10)
474. Carrying Forward the Spirit of China-Arab Friendship and Jointly Building a China-Arab Community with a Shared Futurein the New Era (2022-12-10)
475. Written Remarks by H.E. Wang Yi Member of the Political Bureau of the CPC Central Committee State Councilor and Minister of Foreign Affairs of The People's Republic of China At the Ministerial Meeting of the Group of 77 and China (2022-12-15)
476. Address by H.E. Xi Jinping President of the People’s Republic of China At the Opening of the High-Level Segment of Part II of the 15th Meeting of the Conference of the Parties to the Convention on Biological Diversity (2022-12-16)
477. President Xi Jinping Addresses Opening of High-Level Segment of Part II of COP15 (2022-12-16)
478. Maintain a Global Vision,Forge Ahead with Greater Resolve and Write a New Chapter in Major-Country Diplomacy with Chinese Characteristics (2022-12-25)
479. Remarks by Foreign Minister Qin Gang at the Completion Ceremony for the China-aided Project of the Africa Center for Disease Control and Prevention Headquarters (2023-01-11)
480. Chinese New Year Message to the Diplomatic Corps By H.E. Qin Gang Minister of Foreign Affairs of the People’s Republic of China (2023-01-20)
481. Letter by Wang Yi To the Inauguration Ceremony of the Preparatory Office of the International Organization for Mediation (2023-02-16)
482. Jointly Building the International Organization for Mediation to Establish a New Platform for Peaceful Resolution of International Disputes (2023-02-16)
483. Making the World a Safer Place (2023-02-18)
484. Implementing the Global Security Initiative to Solve the Security Challenges Facing Humanity (2023-02-22)
485. Following a Chinese Path of Human Rights Development and Contributing China’s Strength to Global Human Rights Governance (2023-02-27)
486. Enhancing Multilateralism and Promoting Global Development (2023-03-02)
487. Foreign Minister Qin Gang Meets the Press (2023-03-07)
488. Remarks by Director-General of the Department of Arms Control of the Foreign Ministry Sun Xiaobo at the “Linjia 7 Salon—Press Conference on Japan’s Disposal of the Fukushima Nuclear Contaminated Water” (2023-03-17)
489. Forging Ahead to Open a New Chapter of China-Russia Friendship, Cooperation and Common Development (2023-03-20)
490. Congratulatory Letter by State Councilor and Foreign Minister Qin Gang To the Reception Marking the 78th Anniversary Of the League of Arab States and the 30th Anniversary of the Mission of the League of Arab States in Beijing (2023-03-22)
491. Forging Ahead on the New Journey toward a Community with a Shared Future for Mankind (2023-03-27)
492. Following the Vision of a Community with a Shared Future for Mankind and Bringing More Certainty to World Peace and Development (2023-03-30)
493. Chinese Modernization: New Opportunities for the World (2023-04-21)
494. Providing New Opportunities to the World Through Chinese Modernization (2023-05-16)
495. Written Remarks by H.E. Qin Gang State Councilor and Minister of Foreign Affairs of The People’s Republic of China On UN Commemoration of the 75th Anniversary of the Nakba (2023-05-16)
496. Working Together for a China-Central Asia Community with a Shared Future Featuring Mutual Assistance, Common Development, Universal Security, and Everlasting Friendship (2023-05-19)
497. When Brothers Are of One Heart and One Mind, They Have the Strength to Break Metal (2023-05-25)
498. Remarks by Vice Foreign Minister Ma Zhaoxu at the Launch of the Progress Report on the Global Development Initiative (2023-06-21)
499. Congratulatory MessageFrom State Councilor and Foreign Minister Qin Gang To the Jakarta Forum on ASEAN-China Relations: Commemorating The 20th Anniversary of China’s Accession to the Treaty of Amity And Cooperation in Southeast Asia (2023-06-22)
500. Staying True to Our Founding Mission and Advancing Unity and Coordination to Realize Greater Development (2023-07-04)
